# Degree day-based model predicts pink bollworm phenology across geographical locations of subtropics and semi-arid tropics of India

**DOI:** 10.1038/s41598-020-80184-6

**Published:** 2021-01-11

**Authors:** Babasaheb B. Fand, V. S. Nagrare, S. K. Bal, V. Chinna Babu Naik, B. V. Naikwadi, D. J. Mahule, Nandini Gokte-Narkhedkar, V. N. Waghmare

**Affiliations:** 1 ICAR-Central Institute for Cotton Research, Nagpur, Maharashtra 440 010 India; 2 ICAR-Central Research Institute for Dryland Agriculture, Hyderabad, Telangana 500 059 India

**Keywords:** Ecology, Animal behaviour, Entomology, Agroecology, Behavioural ecology, Climate-change ecology, Ecological modelling, Population dynamics

## Abstract

There is a global concern about the effects of climate change driven shifts in species phenology on crop pests. Using geographically and temporally extensive data set of moth trap catches and temperatures across the cotton growing states of India, we predicted the phenology of cotton pink bollworm *Pectinophora gossypiella* (Saunders). Our approach was centered on growing degree days (GDD), a measure of thermal accumulation that provides a mechanistic link between climate change and species’ phenology. The phenology change was predicted by calculating absolute error associated with DD and ordinal date, an alternative predictor of phenology, for peak moth abundance. Our results show that GDD outperformed the ordinal dates in predicting peak moth abundance in 6 out of 10 selected locations. Using established thresholds of 13.0/34.0 °C, mean DD accumulated between the consecutive moth peaks across different years were estimated at 504.05 ± 4.84. Seven generations were determined for pink bollworm in a cropping season, the length of which varied between 35 and 73 days in response to temperature. Pink bollworm population reached its peak during third generation which can be the target for management actions. The study provides essential information for developing pink bollworm management strategies under climate change.

## Introduction

Recent climate change has markedly shifted the distribution of temperature variability and extremes^1^. These shifts have important ecological consequences for growing environments of most of the organisms, and advancements in the timing of seasonal activities or phenology constitutes high proportion of all the evidences^2–6^. The dynamic nature and diversity of species’ responses to climate change poses significant difficulties for developing robust and long-term management strategies. Temperature plays significant role in phenology of poikilothermic organisms like insects; therefore, understanding their responses to temperature changes is essential to adapt pest management systems under climate change^7,8^. Given that these phenological changes are occurring, an important step is to determine how predictable these changes are especially in the context of changing climate. In these circumstances, the degree-days (DD) seem to be a very strong, integrative and reliable measure of predicting species responses to climate change, because DD accounts for location specific variation in temperature, and it also explicitly impose the thermal limits within which species’ growth is possible^2,9^.

Degree-day models, because of their prediction accuracy over the ordinal dates, are considered as important analytical tools for predicting developmental events in plants and insects^10,11^. Using DD for prediction of insect activity requires estimation of temperature thresholds through the development of temperature-dependent phenology model. The process of phenology model building essentially involves a detailed laboratory investigations on species’ developmental biology at various constant temperatures, and then to estimate the temperature-dependent life processes and developmental thresholds through a fitting of several linear and non-linear mathematical functions of higher biological significance^12–14^. The heat units i.e. DD are then accumulated between the established lower and upper threshold temperatures (LTT and UTT) over the time interval for the developmental event of interest in species’ life history, e.g. development from egg to adult in insects^15,16^.

Vast majority of experimental studies have shown that DD exhibit direct relationship to the development rate of insect larvae^16–20^. These relationships are often used in building temperature-dependent advanced phenology models of certain well-known insect pests, e.g., potato tuber moth *Pthoriemea operculella*^21^, cotton mealybug *Phenacoccus solenopsis* Tinsley^19^, cereal stem borer *Chilo partellus* (Swinhoe)^22^; common cutworm *Spodoptera litura* Fabricious^20^ and cotton pink bollworm *Pectinophora gossypiella*^16^. However, such DD models those are based on the laboratory estimates of developmental thresholds usually need to be calibrated differently for different geographical areas, reflecting the variability in developmental conditions especially the fluctuating rather than constant temperatures in the field relative to laboratory conditions, and the microclimate differences among the evaluated sites and among the individuals of the population^2,17,18^. Field calibration of laboratory estimates of developmental thresholds needs defining the dates of the beginning and the end of the developmental event of interest under a wide range of temperature situations observed over a period of several years. The DD are then accumulated between initial and final dates of the event using a range of combination of lower and upper developmental thresholds. The combination of thresholds which provides the lowest coefficient of variation (CV) of DD between the events offers the best lower and upper thresholds for the species under investigation^10,17,23,24^. Several methods have been advocated for calculating DD through the use of daily maximum and minimum temperatures, e.g. averaging, triangulation and sine wave^25–29^. For use of upper threshold, three different cut-offs are used viz, horizontal cut-off, intermediate cut-off and vertical cut-off depending on whether the rate of development is unchanged, zero or decreasing, respectively^26–28^. The present study used sine wave method with horizontal cut-off for upper threshold as it is most widely accepted among all the methods of DD calculations because of its simplicity and lowest coefficient of variation thus minimising errors in DD estimation^17,25–29^.

Pink bollworm *Pectinophora gossypiella* (Saunders) (Lepidoptera: Gelechidae) is most economically damaging insect pests of global importance to cotton (*Gossypium sp*. Lin.)^30^. This pest has recently become a serious menace on transgenic Bt cotton (both single gene Cry1Ac and dual gene, Cry1Ac + Cry2Ab) in India, causing widespread damage and approximate yield losses to the tune of 20–30%^31,32^. The re-emergence of pink bollworm has serious ecological and economic implications for cotton production, especially in the context of impending climate change. Considering this, the temperature rise of 2.7–4.7 °C predicted due to potential climate change^4^ may have drastic consequences for future invasiveness of pink bollworm. In this context, understanding how pink bollworm will respond to temperature changes is crucial to devise future climate resilient management strategies for this pest. Several studies have proposed DD models to describe pink bollworm development^16,17,25,33,34^. However, majority of these models (except Beasley and Adams^17^) are based on laboratory studies conducted at constant temperatures. Further, spatio-temporal variability in temperature and microclimate impose restrictions on blanket use of developmental thresholds across geographical locations that reflect wide bioclimatic variability^10,26^. In our previous study^16^, we estimated developmental threshold temperatures of 13.4 °C/35.5 °C and thermal requirements of 503.62 DD for pink bollworm through temperature-dependent phenology model fitted to the laboratory data on its development at different constant temperatures between 15 and 38 °C. The estimated threshold temperatures accumulated the heat units closer to the laboratory estimates and sensibly predicted the developmental events in pink bollworm under field conditions. However, there is limited applicability of laboratory based thresholds in accurately predicting the development under field conditions that are characterised by temperature and microclimate variability^2,17,18,35^.

The research reported here was prompted by the paucity of developmental thresholds obtained from field data for predicting pink bollworm development, and the lack of deeper insights into its bio–ecology and population growth potential under Indian scenario of cropping environments and cultural practices. The fundamental objectives of the present study were: (1) To calibrate the laboratory estimates of developmental thresholds for field prediction, (2) To estimate the generation events in a seasonal cycle based on DD accumulations between successive moth trap catch peaks coupled with host crop phenology, (3) To determine the predictive ability of DD versus ordinal date in predicting pink bollworm phenology using long term data on pheromone trap catches and daily temperatures, and (4) To understand the ecobiological behaviour of pink bollworm in different climatic zones of India in order to develop predictive models of its population dynamics and seasonality by using time series analysis. The salient findings of this research demonstrate that more accurate prediction of pest activity can be achieved by using thermal summation (growing degree day, GDD) approach coupled with a data on host crop phenology and indicates that accurate estimates of how climate change will influence pest’s phenology require explicitly accounting for variation in temperature.

## Results

### Field calibration and validation of developmental thresholds

**Figure 1:**
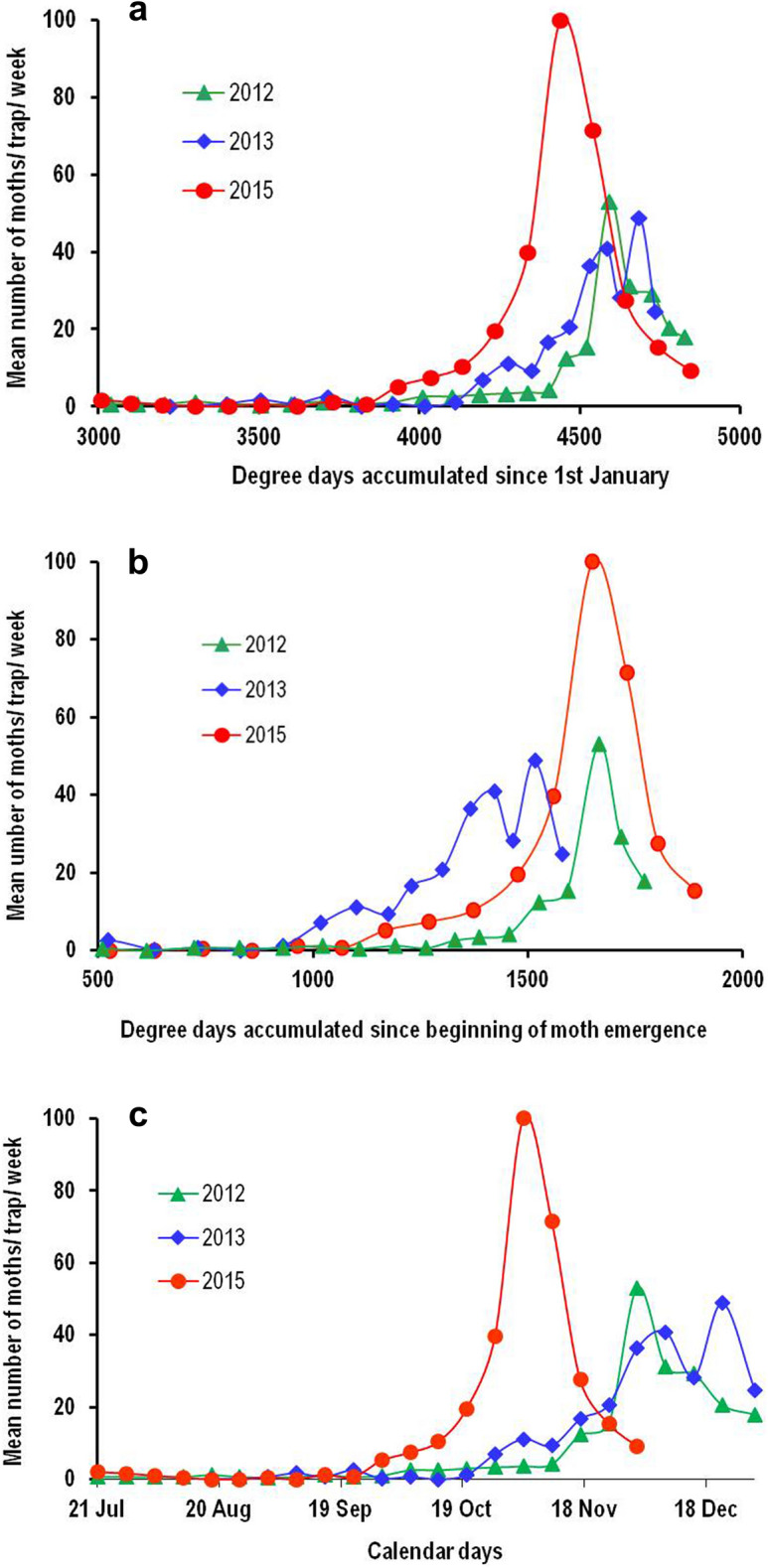
Mean number of pink bollworm moth catches per trap per week recorded from sex pheromone traps installed along the periphery of cotton fields in the experimental farm of ICAR-CICR, Nagpur (India) during 2012–2015. Coincidence or overlapping of moth peaks when plotted on degree-day scale (**a**,**b**), Non-overlapping of moth peaks when plotted on calendar date scale (**c**).

The lower and upper developmental threshold temperatures for pink bollworm selected based on lowest CV of DD accumulated by single sine wave method with upper horizontal cut-off were 13.0 °C and 34.0 °C, respectively. Using these thresholds, the CV obtained for DD accumulations from 1st January to beginning of moth emergence and beginning of emergence to next successive moth catch peak were 5.53% and 1.00%, respectively as against CV values of 5.66% and 1.27% obtained with laboratory estimates of thresholds (Table 1). The mean DD accumulated from 1st January to beginning of moth emergence using 8 years moth catch data of Nagpur (Maharashtra state) were 3073.55 ± 60.09 DD. Similarly, mean DD accumulated between beginning of moth emergence and next successive moth catch peak were 506.54 ± 1.66 DD which is approximate to one in-field generation of pink bollworm. The field selected thresholds of 13.0 °C and 34.0 °C provided reasonably stable estimates of DD accumulations for both the events, which was evident from fair overlapping of peaks of moth catches during different years (2012–15) when moth catch data was plotted versus DD (Fig. 1a,b). On the other hand, there was a clear mismatch between the moth catch peaks across these years when plotted against calendar dates (Fig. 1c).

**Table 1:**
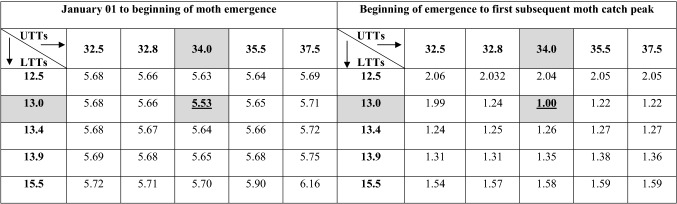
Field calibration of optimum developmental threshold temperatures for cotton pink bollworm using coefficients of variation (CV) technique of degree-day accumulations calculated for male moth catches in sex pheromone traps observed for a period of 8 years using different combinations of lower and upper threshold temperatures.

A combination with lowest CV is accepted as the best lower and upper threshold temperatures.

Bold underline values represent the best combination of LTT and UTT (13.0/34.0 °C) which gave lowest value of coefficient of variation in degree days estimation.

The threshold combination of 13.0/34.0 °C selected as optimal developmental threshold temperatures for predicting pink bollworm development under field condition provided relatively closer estimates of DD accumulations between successive peaks of moth trap catches. The trap catch data used for DD estimation were collected between 2007–16 at Faridkot (Haryana), Junagadh (Gujarat) and Dharwad (Karnataka) locations representing wide variability in diurnal and interannual temperatures from north, central and south cotton growing zones of India. The DD accumulations between successive moth catch peaks ranged between 496.20 to 512.00 DD, representing a deviation of − 7.42 to + 8.42 DD which was equivalent to ± 1.0 day. On the other hand, the number of days lapsed between these successive events (equivalent to one in-field generation) varied from 27 to 59 in response to temperatures (Table 2).

**Table 2:** Validation of degree-day model of cotton pink bollworm in three different cotton growing zones of India.

Year	Faridkot, Haryana				Junagadh, Gujarat				Dharwad, Karnataka			
	Start date	End date	DD	Days	Start date	End date	DD	Days	Start date	End date	DD	Days
2007–08	02 Jul	28 Jul	510.5	27	–	–	–	–	29 Jul	13 Sept	501.9	47
	28-Jul	24 Aug	499.5	27	–	–	–	–	13 Sept	28 Oct	506	45
	24 Aug	22 Sep	508.0	29	–	–	–	–	28 Oct	23 Dec	503.6	56
	22 Sep	30 Oct	503.0	38	–	–	–	–	–	–	–	–
2008–09	16 Jul	14 Aug	506.3	30	–	–	–	–	12 Nov	08 Jan	503.1	58
	14 Aug	19 Sep	507.2	34	–	–	–	–	–	–	–	–
	19 Sep	02 Nov	501.6	44	–	–	–	–	–	–	–	–
2009–10	23 Jul	18 Aug	507.1	27	–	–	–	–	15 Oct	30 Nov	507.7	47
	18 Aug	19 Sep	511.6	32	–	–	–	–	30 Nov	25 Jan	498.7	56
	19 Sep	20 Oct	504.4	31	–	–	–	–				
2010–11	–	–	–	–	–	–	–	–	29 Jul	13 Sep	504.1	47
	–	–	–	–	–	–	-	–	13 Sep	02 Nov	510.7	50
	–	–	–	–	–	–	–	–	02 Nov	28 Dec	503.8	56
2011–12	23 Jul	24 Aug	507.8	33	24 Sep	25 Oct	501.1	32	–	–	–	–
	24 Aug	26 Sep	510.8	33	25 Oct	29 Nov	502.6	35	–	–	–	–
	26 Sep	29 Oct	507.7	32	29 Nov	27 Jan	504.4	59	–	–	–	–
2014–15	–	–	–	–	06 Aug	10 Sep	512.0	36	–	–	–	–
	–	–	–	–	10 Sep	13 Oct	505.5	34	–	–	–	–
	–	–	–	–	13 Oct	19 Nov	507.4	36	–	–	–	–
	–	–	–	–	19 Nov	16 Jan 15	501.3	58	–	–	–	–
2015–16	01 Jul	29 Jul	499.7	29	09 Jul	09 Aug	510.6	32	–	–	–	–
	29 Jul	27 Aug	506.9	29	09 Aug	10 Sep	508.0	32	–	–	–	–
	27 Aug	28 Sep	508.9	32	10 Sep	11 Oct	496.2	31	–	–	–	–
	–	–	–	–	11 Oct	14 Nov	507.2	34	–	–	–	–
	–	–	–	–	14 Nov	01 Jan	508.8	48	–	–	–	–

The degree days were accumulated between successive moth catch peaks starting from beginning of moth emergence for each year at each location.

### Estimation and validation of in-field generation events

**Figure 2:**
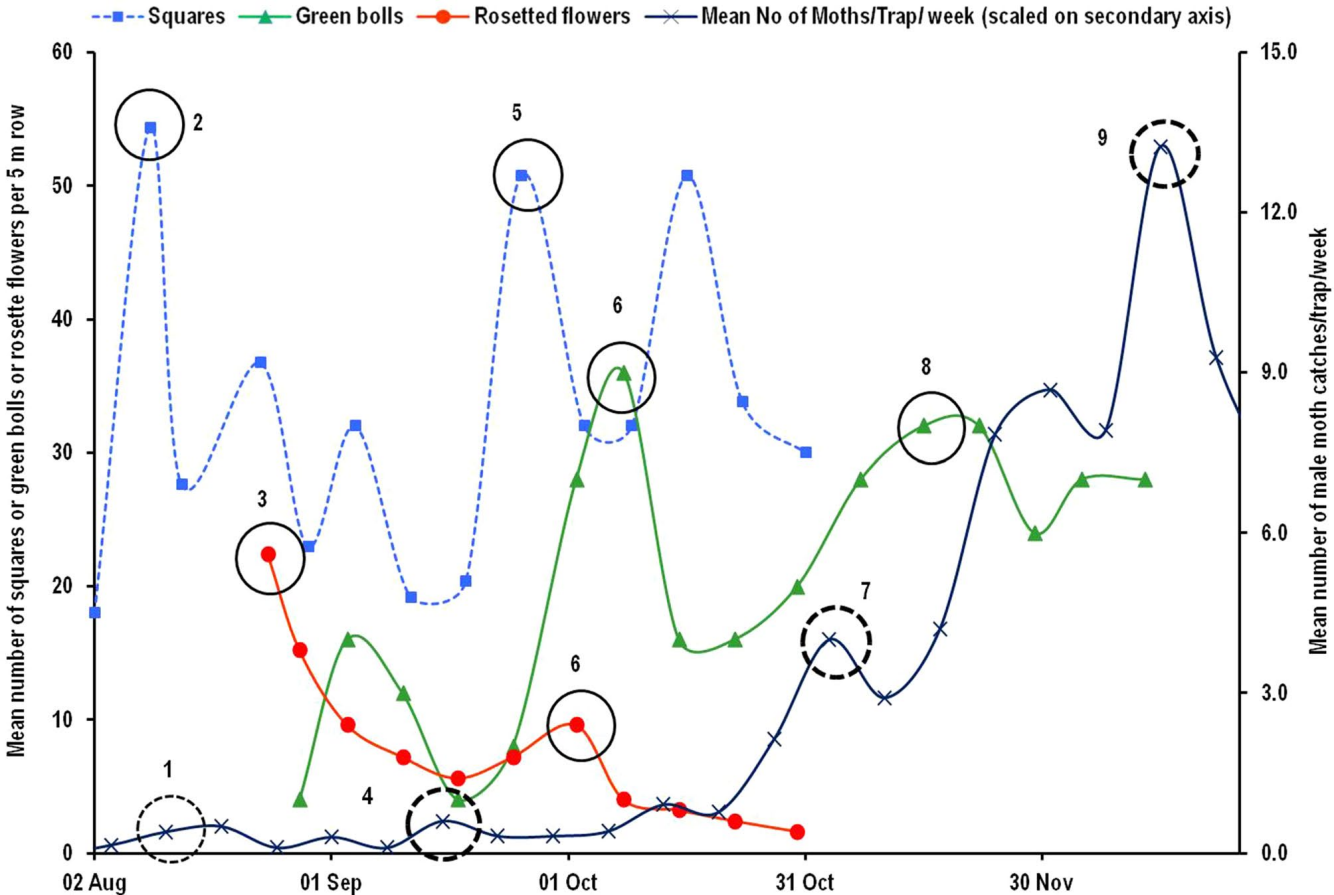
Estimation of number of generations completed by pink bollworm in a cotton season based on DD accumulations between successive male moth catch peaks obtained in sex pheromone traps during the year 2018–19 at Nagpur (India). The DD accumulations were started from 2 weeks prior to rosette flower appearance in field. The dotted circle no. 1 denotes the availability of male moths to mate with female moths to oviposit on susceptible squares in circle no. 2 which gave rise to rosetted flowers approx. 2 weeks later as indicated in circle no. 3 from which emerged the first flush of moths (F1) produced within current season’s cotton crop, indicated by circle no. 4. The cycle is repeated and giving rise to F2 and F3 moths as indicated in circle nos. 7 and 9.

**Figure 3:**
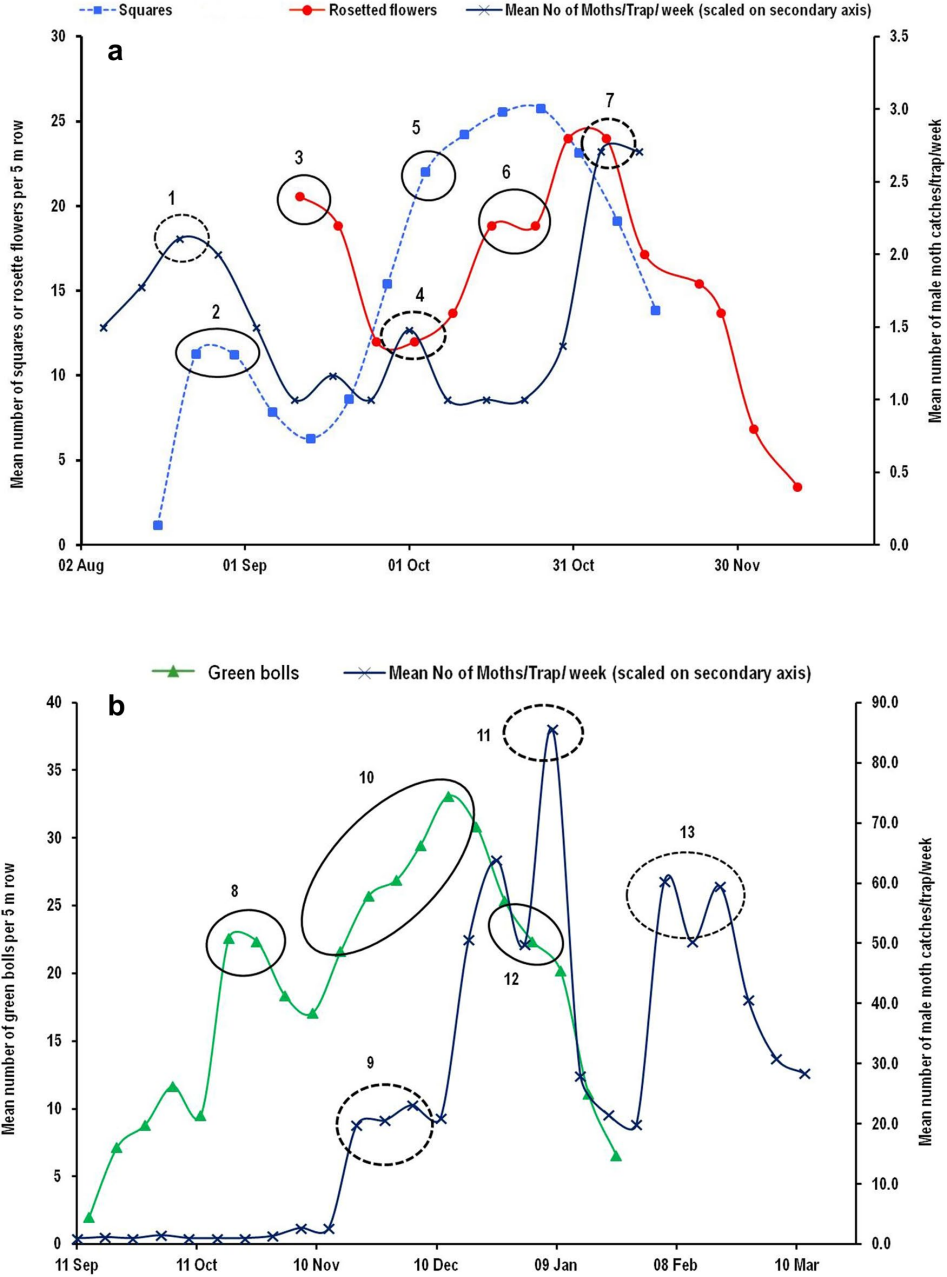
Validation of generation events of pink bollworm during the cotton season 2019–20. Till November 10, moth catches were very low (< 3 moths per trap per week) and pink bollworm could complete two in-field generations as indicated in dotted circle nos. 4 and 7 (**a**); whereas with ample availability of green bolls, the pest’s preference shifts from flowers to bolls giving rise to three in-field generations as indicated by dotted circle nos. 9, 11 and 13 (**b**).

**Figure 4:**
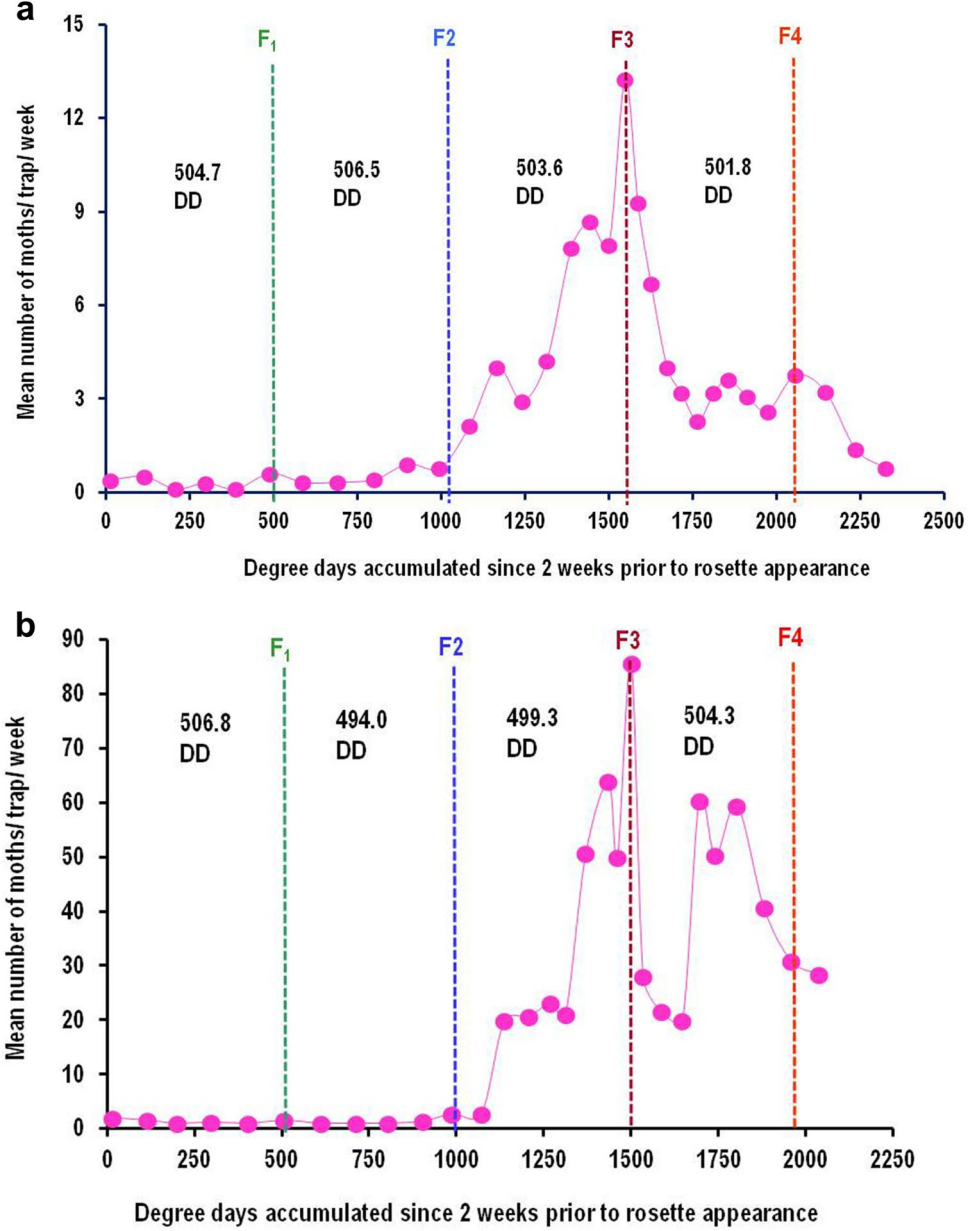
Generation events estimated for pink bollworm based on degree-day accumulations between successive moth catch peaks recorded in sex pheromone traps at Nagpur (India). When moth catches were plotted against DD accumulations, the number of DD required for completing one generation remained fairly constant in a range between 501.8–506.5 during 2018–19 (Fig. 5a), and 494.0–506.8 during 2019–20 (Fig. 5b).

**Figure 5:**
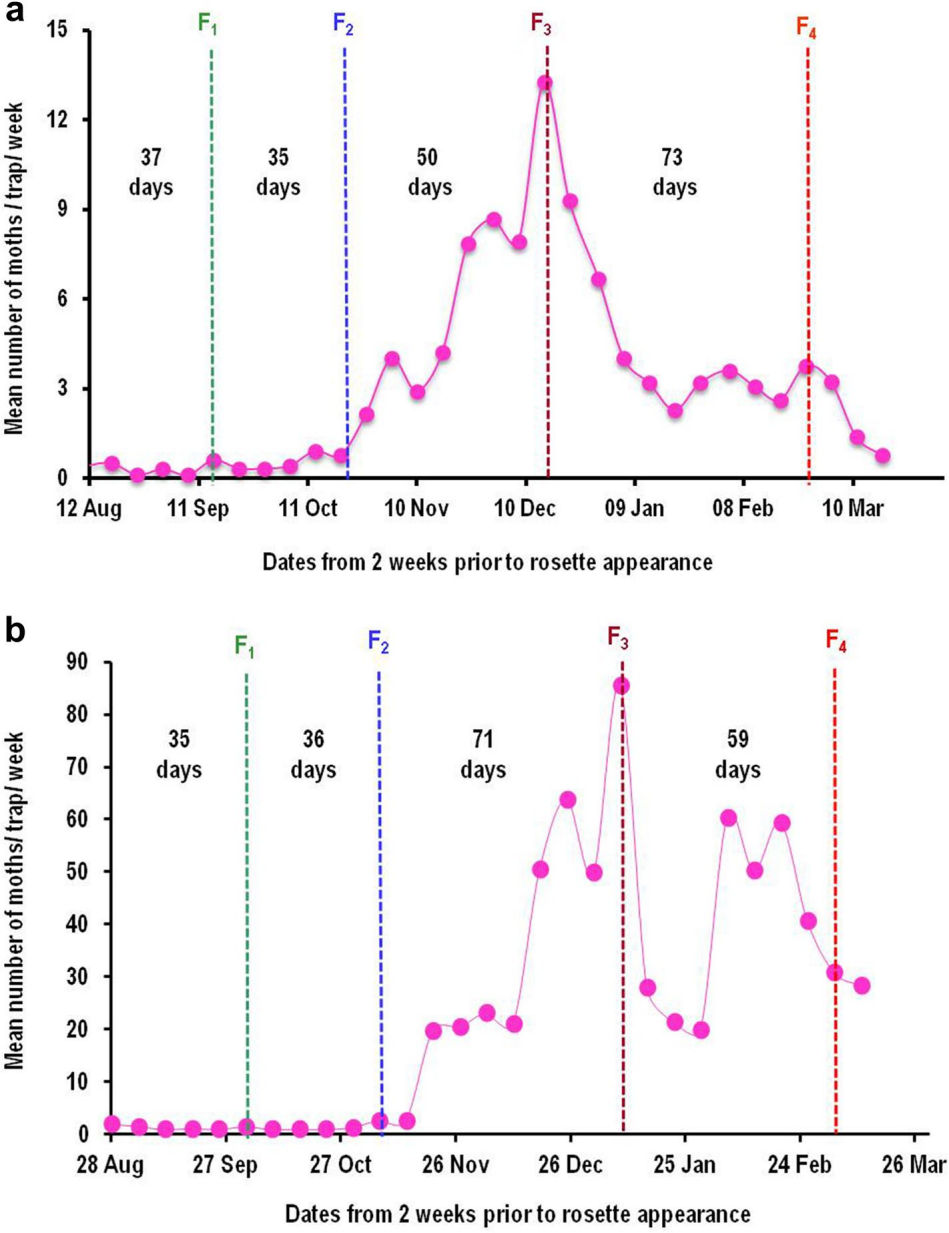
Generation events estimated for pink bollworm based on days lapsed between successive moth catch peaks recorded in sex pheromone traps at Nagpur (India). When moth catches were plotted against calendar dates, the number of days per generation varied between 35–73 during 2018–19 (Fig. 6a) and 35–71 during 2019–20 (Fig. 6b) in response to changes in daily minimum and maximum temperatures.

The data on male moth captures in sex pheromone traps recorded in test fields at Nagpur (Maharashtra state) were related to the appearance of susceptible squares, rosette flowers and damaged green bolls as a result of pink bollworm infestation during two cotton growing seasons of 2018–19 (Fig. 2) and 2019–20 (Fig. 3a,b). The DD accumulations starting from two weeks prior to appearance of rosette flowers provided reliable estimates of the generation events during both the cropping seasons. The initial dates for DD accumulations (biofix) were 12th August and 28th August for the cropping seasons of 2018–19 and 2019–20, respectively. Using these biofix dates, we could determine up to four non-overlapping in-field generations of pink bollworm. Initial two generations could easily be determined whereas; it was relatively difficult to determine third and fourth generations because of frequent overlapping of moth peak curves and steep increase in number of moths captured in later part of the season, making it complicated to determine exact peak date^17^. However, DD accumulations between the peaks helped us in tracing the possible dates of peak captures for later generations. The intermittent peaks not matched by estimations of generation events may constitute overlapping generations of pink bollworm from continuously emerging moths (Figs. 4 and 5). Thus, about seven generations (including four non-overlapping and three overlapping generations from intermittent peaks) can be completed by pink bollworm if crop season extends from Mid June to as late as mid February or first week of March under Central Indian conditions. The DD accumulated using sine wave method for one in-field generation of pink bollworm, based on F_1_ to F_4_ generation events ranged between 501.8–506.5 and 494.0–506.8 for the crop seasons of 2018–19 and 2019–20, respectively. These values were closer to the value of 503.62 DD estimated for pink bollworm development from egg to adult based on our previous temperature dependent laboratory study^16^. On the other hand, fairly distant values of 489.90–497.90 DD were obtained when laboratory-based threshold combination (13.4/35.5 °C) was used for DD accumulations using same data. Thus, field calibrated developmental thresholds (13.0/34.0 °C) reduced the margin of error from ± 9.72 DD to ± 2.34 DD in terms of predicted DD and ± 1.0 day to ˂ ± 0.5 day in terms of predicted days for the same field data at same location. The pattern of moth captures for the generation events of F_1_ to F_4_ remained relatively consistent during both the years when plotted against DD scale (Fig. 4a,b). However, the number of days per generation varied from 35 to 73 and 35 to 71 in response to variations in seasonal temperatures during the cotton cropping seasons of 2018–19 and 2019–20, respectively (Fig. 5a,b).

### Predictive ability of DD vs ordinal date for predicting pink bollworm phenology

Six out of 10 selected locations had significant predictions of peak moth catch dates using DD method than ordinal date. Significantly, a greater number of site-year combinations with less prediction error were associated with DD compared to ordinal date (Table 3). Thus, DD outperformed ordinal date and more accurately predicted peak moth catches for pink bollworm in 48 of 51 survey years. On the other hand, date was marginally significantly better in predicting peak moth catches for few site-year combinations. There were no site-year combinations present where DD and date had equal prediction errors. The time series analysis with ARIMA model indicated that DD performed significantly better than date and predicted more accurately the peak moth catch dates of pink bollworm across all the selected 10 locations (Tables 4 and 5).

**Table 3:** Number of site-year combinations for pink bollworm showing less or equal prediction errors for peak moth catches based on growing degree days (GDD) or calendar dates when compared to a mean value based on n-1 years at each site (Chi^2^ test applied @ df = 1).

S.N	Site/location	Number of years with less prediction error		Number years with equal prediction error	*P* value
		GDD	Date		
1.	Faridkot	7	1	0	< 0.00001**
2.	Sriganganagar	6	4	0	0.317
3.	Junagadh	3	1	0	0.045*
4.	Surat	6	1	0	< 0.00001**
5.	Nagpur	8	5	0	0.179
6.	Rahuri	4	1	0	0.0027**
7.	Dharwad	4	1	0	0.0027**
8.	Raichur	2	2	0	1.00
9.	Lam, Guntur	2	1	0	0.317
10.	Nandyal	6	2	0	0.005**
11.	Total site-year combinations	48	19	0	< 0.00001**

*Chi^2^ value significant at 0.05% level of significance.

** Chi^2^ value highly significant at 0.05% level of significance.

**Table 4:** Predicted GDD or dates of peak moth catches of pink bollworm by ARIMA model.

S.N	Site/location	GDD			Date		
		Observed	Predicted by ARIMA model	Residual DD	Observed	Predicted by ARIMA model	Residual days
1	Faridkot	1520.00	1531.22	11.22	26 Sep	10 Oct	15.00
2	Sriganganagar	1558.60	1514.51	44.09	21 Oct	14 Oct	7.00
3	Junagadh	1525.60	1520.98	4.62	18 Oct	20 Dec	63.00
4	Surat	1566.30	1579.85	13.55	07 Nov	25 Nov	18.00
5	Nagpur	1519.50	1518.50	1.00	16 Dec	05 Dec	11.00
6	Rahuri	1498.50	1484.30	14.20	25 Nov	23 Dec	28.00
7	Dharwad	1516.10	1515.08	1.02	10 Dec	16 Dec	6.00
8	Raichur	1517.60	1517.45	0.15	13 Jan	11 Jan	1.50
9	Lam, Guntur	1538.95	1527.03	11.92	25 Dec	07 Jan	13.50
10	Nandyal	1530.700	1532.900	2.20	31 Dec	24 Dec	7.00

**Table 5:** Predictive ability of GDD vs calendar dates in forecasting the dates of peak moth emergence as revealed from ARIMA model applied to the trap catch data for all the years at each location.

S. no.	Site/location	Peak emergence of pink bollworm			Residuals (days)	
		Observed dates	Dates predicted by ARIMA model	Dates converted from GDD predicted by ARIMA model	Based on dates	Based on GDD
(A)	(B)	(C)	(D)	(E)	(C-D)	(C-E)
1.	Faridkot	26 September	10 October	26-September	− 15.00	0
2.	Sriganganagar	21 October	14 October	18-October	7.00	3
3.	Junagadh	18 October	20 December	18-October	− 63.00	0
4.	Surat	07 November	25 November	08-November	− 18.00	-1
5.	Nagpur	16 December	05 December	16-December	11.00	0
6.	Rahuri	25 November	23 December	24-November	− 28.00	1
7.	Dharwad	10 December	16 December	10-December	− 6.00	0
8.	Raichur	13 January	11 January	12-January	1.50	1
9.	Lam, Guntur	25 December	07 January	03-January	− 13.50	− 9
10.	Nandyal	31 December	24 December	01-January	7.00	− 1

**Figure 6:**
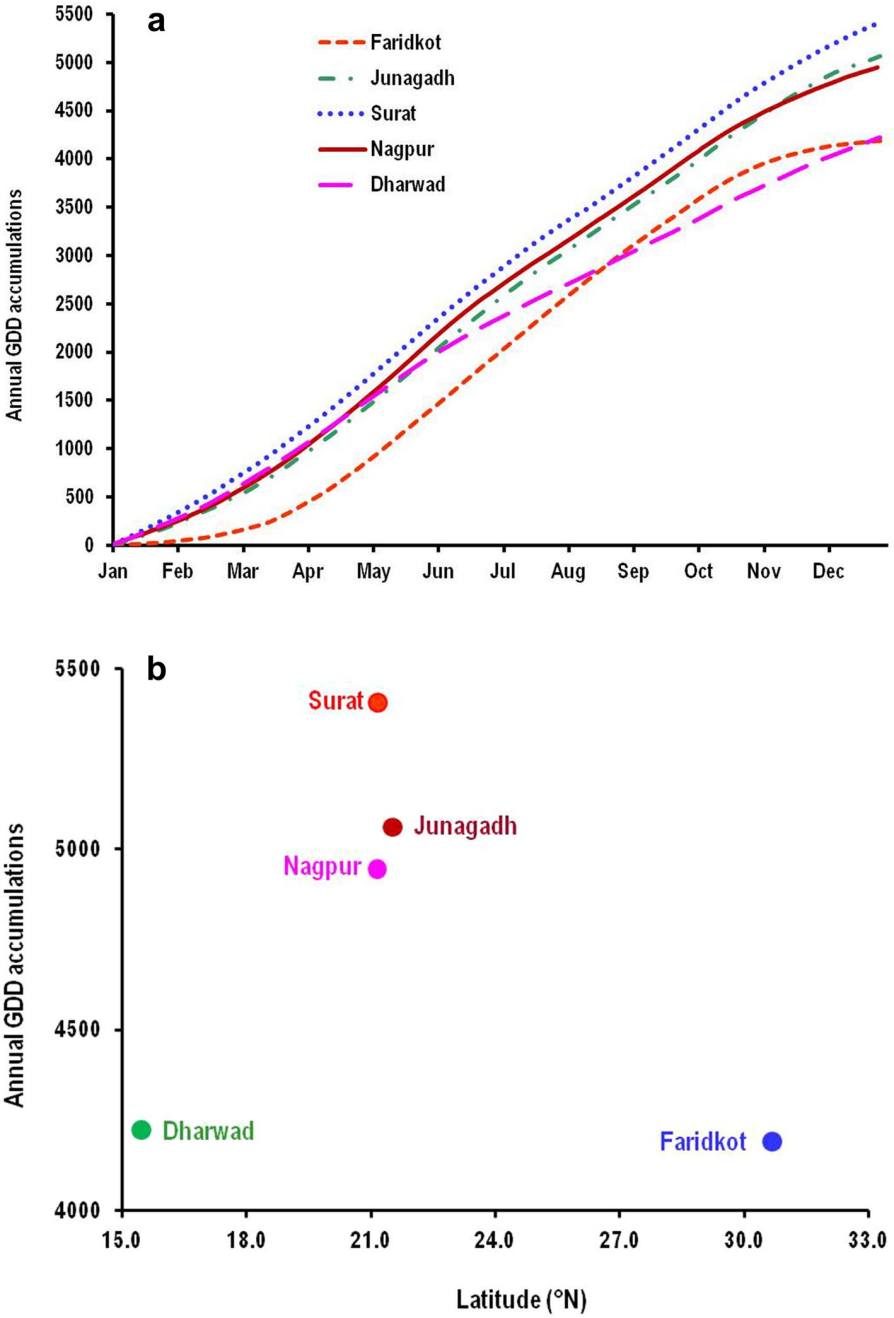
Mean annual growing degree days (GDD) accumulated at each sampling sites averaged over all the study years (**a**) and latitudinal variation in mean GDD accumulation for different sampling sites ordered by latitude from south to north (**b**).

**Figure 7:**
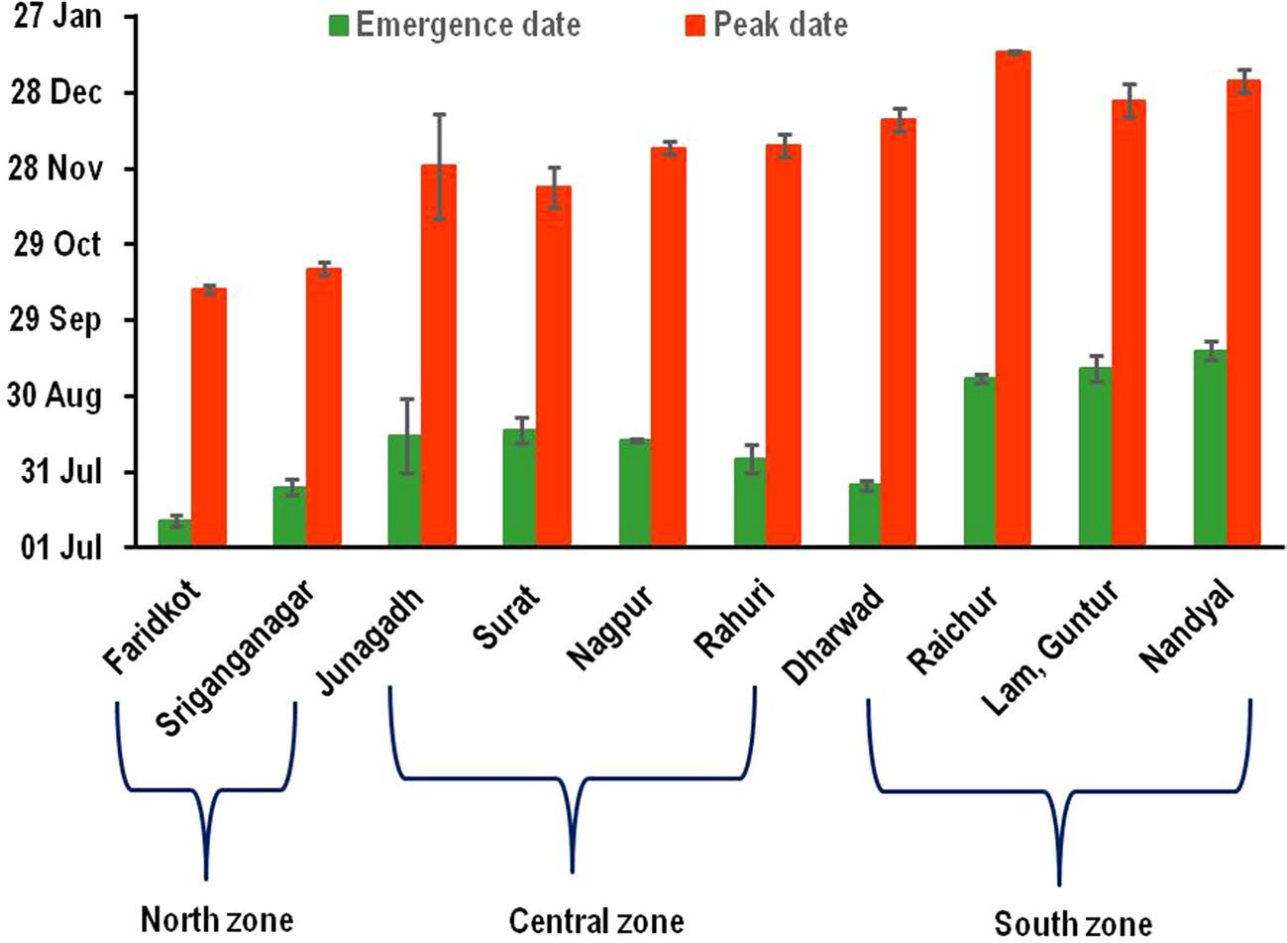
A progressive shift in the dates of beginning of emergence and peak moth catches observed from north to south cotton growing locations of India.

The interannual variation in DD accumulations across different geographical locations was revealed by mean annual DD accumulated at five out of 10 selected locations. Annual accumulations of DD averaged among all the years at each site ranged from lowest value of 4189.94 ± 54.9 DD at Faridkot to highest value of 5407 ± 173.1 DD at Surat (Fig. 6a). High level of latitudinal variation from south to north was observed in annual DD accumulations (Fig. 6b). A progressive shift with an approximate monthly lag in the dates of beginning of emergence and peak moth catches was observed from north to south (Fig. 7). On an average, the moth emergence started in the first week to the middle of July, August and September months in north, central and south Indian locations, respectively, only the exception was Dharwad from south zone where the emergence started in July. The pink bollworm population reached at its peak as revealed from moth catches during the mid of October (North zone), end of November to first week of December (Central Zone) and December end to first week of January (South Zone). The mean DD accumulation to peak moth catches averaged over all the years at each of the 10 selected locations ranged between 1501.8 ± 9.32 to 1553.45 ± 12.10. Considering the mean DD requirement of approximately 500 ± 10 for one generation of pink bollworm, we could determine three in-field generations from beginning of moth emergence to peak moth catch date. Accordingly, the DD accumulations per generation for these 10 locations ranged between 500.60 and 517.82.

## Discussion

Comprehensive, DD based phenology model is presented for predicting pink bollworm developmental events under field conditions that are characterised by wide spatio-temporal variability in temperature and microclimate. The non-coincidence of the moth catch peaks observed across the study years when plotted against calendar dates may be because of a warmer than usual summer in the year 2015 that has advanced the emergence and peak abundance of pink bollworm compared to rest of the two years 2012 and 2013. However, coincidence of moth trap catch peaks across the years when plotted on DD scale confirmed that the thresholds selected based on lowest CV of DD accumulations between the events were optimal enough to describe field development of pink bollworm. The developmental thresholds of 13.0/34.0 °C derived herein using field data had improved predictive ability of pink bollworm developmental events compared to the laboratory thresholds of 13.4/35.5 °C reported in our previous study^16^. This was evident from the reduced margin of error for predicted DD between two successive moth emergence peaks. This entails that our choice seems to be appropriate for single sine wave method with upper horizontal cut-off for DD accumulations, a most widely accepted method that provides lowest CV and reduces the margin of prediction error^17,27–29^. The total heat unit requirement estimated using these thresholds for completion of one in-field generation of pink bollworm was in line with the various literature reports, e.g. 503.62 DD^16^, 492.00 DD^17^, 499.90 DD^36^, 499.13–522.11 DD^37^ and 502 DD^38^.

The validation results indicated that, the developmental threshold temperatures presented here are useful for relating daily minimum and maximum temperatures to pink bollworm development in cotton fields across different geographical locations. The margin of error of ± 1.0 day estimated in present study for predicting peaks of moth trap catches from DD accumulations based on daily temperature data at different test locations is fairly reasonable to use in field predictions. The slight deviations in the DD estimated in present study and those reported in literature may be due to source of temperature data used in DD estimations. We used daily minimum and maximum temperatures for most of the locations, whereas for few locations, monthly mean values of the same were used because of non-availability of daily data, which could be one of the possible sources of variation. Further, these were the values of air temperatures, which may not be identical to the microclimate i.e. temperatures within cotton canopy, squares and bolls experienced by pink bollworm under field condition^17,39^. Despite minor variations in estimated and reported DD accumulations, it is encouraging that the combination of thermal thresholds presented herein, when applied to field data, satisfactorily predicted the peaks of pink bollworm moth emergence across geographically and climatically diverse locations.

Our results of seven in-field generations determined for pink bollworm are fairly in agreement with the literature reports on number of generations of pink bollworm in a cotton cropping season, e.g. 4–6 generations are reported in North India where the pest enters early diapauses due to severe winter and the crop is terminated well before November, and 8–9 generations in Central and South India where winters are relatively warmer and crop season is generally extended till March-April^40^, five generations reported in California^41^, 5–6 generations in Israel^42^, and 4–6 generations in USA^30^.

In India, pink bollworm is generally a late season pest of cotton crop, the infestation of which begins from middle of the crop season with onset of fruiting structures, and increases steadily towards end of the season^43,44^. This is confirmed by the steep increase in number of moths captured in pheromone traps towards the end of the season as seen in the Figs. 4 and 5, indicating increased severity of damage. One of the important strategies of pink bollworm management in central cotton growing zone of the country is ‘*timely termination of crop*’ either by December end or latest by mid January^27^. Our results presented here indicates that pink bollworm can complete only five generations, if crop season is ended during late December–early January, thus two in-field generations (one non-overlapping and one from overlapping peaks) could be prevented and possible yield loss can be minimised by adopting timely crop termination.

The results of present study showed DD had improved predictions of pink bollworm phenology in responses to spatio-temporal variations in temperatures, as evident from the lower error based on predicted and observed values for DD compared to ordinal date. The predictive ability of DD over ordinal date may be ascribed to its mechanistic link between thermal environment and organism’s development which is lacking in case of ordinal date^9^. A strong trend of improved predictions of peak moth catches observed with DD compared to ordinal date was reflected in time series analysis using ARIMA model. The greater predictability of DD compared to ordinal date for pink bollworm moth catches may further be due to monophagous nature of this pest species with cotton as its only food plant. The outperforming nature of DD over ordinal date in predicting species phenology had been reported for the species with greater dietary specialization i.e. narrow host range^9^. With an average interval of 8 days between surveys, exact dates of emergence and peak catches of pink bollworm moths were not expected, but at least a reasonable estimate of them were obtained given the challenges of collecting large-scale data over a long period of time.

Time series analysis of peak moth catches data of pink bollworm across 10 selected locations revealed that the pest population reached at its seasonal peak mostly in its third generation at all the test locations. This has important implications from management point of view where maximum damaging population can be targeted for effective control and for avoiding economic yield loss to the cotton. Our findings are supported strongly by the studies on bioecology of pink bollworm in relation to cotton phenology^41,45,46^. The squares, blooms, and bolls of cotton plants are the preferred feeding sites of pink bollworm. Usually, the first in-field generation of pink bollworm is completed on squares and flowers, whereas second generation onwards are completed on green bolls^47^. The moths emerging from overwintering population, after mating start laying eggs on young floral buds i.e. squares. The larvae feed and develop within squares leading to formation of rosette flowers, and pupate either in rosette flowers or in soil debris near plant base. The chain of events is repeated for subsequent generations with paradigm shift in larval preference for feeding site from squares to green bolls^41,47^. Thus, the cotton plant becomes a favourable host for pink bollworm infestation due to availability of flowering and fruiting structures approximately from 40 DAS. Accordingly, we can hypothesize that pink bollworm colonizing cotton fields at the squaring stage is expected to complete a generation (lasting for 30–35 days) at approximately 70–80 DAS of crop, and develop a second generation during the predominance of boll formation from 80 to 100 DAS, whereas a third generation may occur during the boll maturation stage (≥ 120 DAS). Therefore, development of a large population of pink bollworm would not be expected early in the cotton season, as only squares would be available for feeding. Low survival rate of pink bollworm on squares compared to bolls have been reported due to increased larval mortality as a result of square shedding^46^. Thus, the large outbreak or peak abundance is expected to occur only after second generation within cotton fields. We observed peak abundance during third generation in all the test locations. This can be the best target for undertaking management actions.

Location specific variation in the dates of beginning of emergence and peak moth catches across north, central and south cotton growing zones of India indicated that in addition to temperature, the other abiotic (microclimate) and biotic (physiological response of species, predation, parasitism) factors do play a significant role in determining pink bollworm phenology. This has several ecological implications in terms of pink bollworm responses to climate change as the pest has restricted range of food plants. The temperature rise under future climate change and associated rapid rates of thermal accumulations have been reported to cause phenological shifts in terms of advancement of emergence and peak abundance dates most dramatically in species with greater dietary specialization^9,48^.

The present study has generated very useful ecological information critical to the management of pink bollworm. Our study show that DD is a good predictor of pink bollworm phenology in terms of forecasting the dates of beginning of emergence and peak abundance, indicating its great potential to be applicable in ecological studies. This framework of DD can be a promising tool to predict the phenological responses of pink bollworm in the context of global temperature rise. The approach can readily be applicable to many other economically important agricultural insect pests outside the scope of present analysis.

## Methods

### Pink bollworm moth catches data

**Figure 8:**
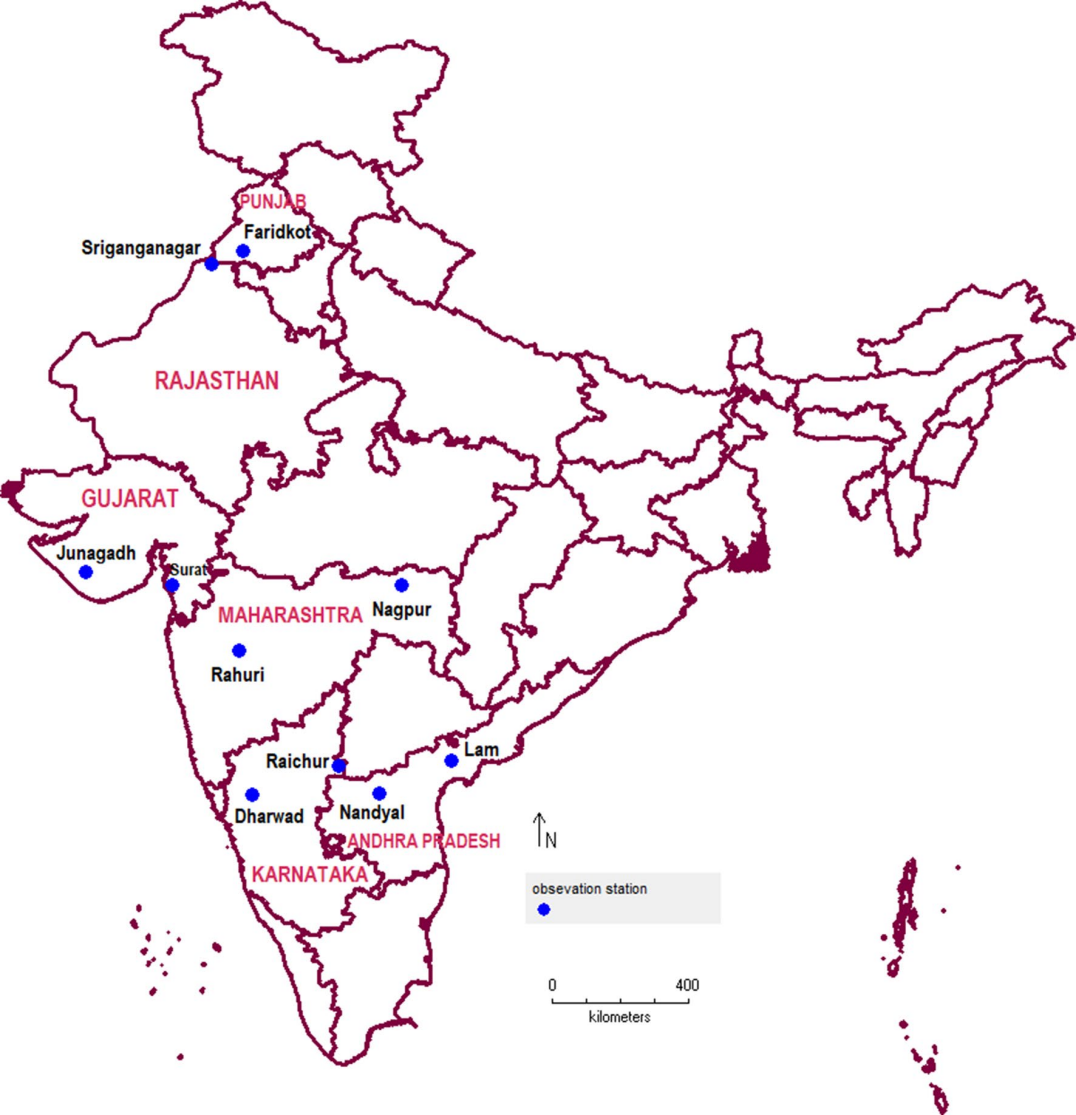
The map of India showing the observation stations and study area.

Ten different locations from north (02), central (04) and south (04) cotton growing zones of India with diverse geographical and bioclimatic features were selected in present study (Table 6, Fig. 8). We obtained survey data on pink bollworm moth catches in sex pheromone traps for these locations. The data were collected between 2006 and 2019 by ICAR-All India Coordinated Research Project on cotton (ICAR-AICRP on cotton) head quartered at Coimbatore in Tamil Nadu state of India through its coordinating centres distributed throughout the country. This data comprised of nine out of ten selected locations, whereas the data for Nagpur location was recorded by the authors of present study. In all cases, the surveys typically run from early June until late February or early March in few exceptions, and were conducted at approximately one-week intervals. The details of the data set used for present analyses is given in Table 7.

**Table 6:** Characteristic features of sampling sites in relation to pink bollworm bioecology.

Zone	North India		Central India				South India			
Locality	Faridkot	Sriganganagar	Junagadh	Surat	Nagpur	Rahuri	Dharwad	Raichur	Lam, Guntur	Nandyal
State	Haryana	Rajasthan	Gujarat	Gujarat	Maharashtra	Maharashtra	Karnataka	Karnataka	Andhra Pradesh	Andhra Pradesh
Coordinates	30.68° N 74.76° E	29.91° N 73.88° E	21.52° N 70.46° E	21.17° N 72.83° E	21.15° N 79.09° E	19.39° N 74.65° E	15.46° N 75.01° E	16.22° N 77.36° E	16.37° N, 80.43° E	15.48° N 78.48° E
Altitude (m)	196	178	107	13	310	511	751	407	33	215
Mean annual temperature (°C)	24.1	24.9	25.7	27.2	26.9	25.9	24.3	27.7	28.5	28.4
Mean annual rainfall (mm)	445	261	827	1192	1092	511	885	713	906	707
Bioclimatic conditions	Sub-tropical arid	Sub-tropical arid	Semi-arid tropics	Semi-arid tropics	Semi-arid tropics	Semi-arid tropics	Semi-arid tropics	Semi-arid tropics	Semi-arid tropics	Semi-arid tropics
Cotton cropping season	April–May to October–November	April–May to October–November	June to December–January	June to December–January	June to December–January	June to December–January	July–August to January–February	July–August to January–February	July–August to January–February	July–August to January–February

**Table 7:** Locations wise details of weather data and pink bollworm moth trap catches data used for analysis in present study.

S.N	Location	Period of data used	Number of years
1.	Faridkot	2007–09, 2011–12, 2015, 2017	07
2.	Sriganganagar*	2012–15, 2017–18	06
3.	Junagadh	2011, 2014–15	03
4.	Surat	2006, 2012, 2014–16, 2018	06
5.	Nagpur	2009, 2012–2019	09
6.	Rahuri*	2013, 2015, 2017–18	04
7.	Dharwad	2006–07, 2010, 2018	04
8.	Raichur	2016–18	03
9.	Lam, Guntur*	2016–18	03
10.	Nandyal*	2012–13, 2015–18	06
Total number of surveys			51

The locations with availability of minimum three years of both data sets were only selected.

*Monthly mean values of minimum and maximum temperatures were used.

### Weather data

The data on daily minimum and maximum temperatures for four locations viz., Junagadh, Surat, Dharwad and Raichur were obtained from the climate database of ICAR-All India Coordinated Research Project on Agrometeorology (ICAR-AICRPAM) Located in the city of Hyderabad in Telangana state of India. The daily temperature data for Nagpur location was collected from Agrometeorological observatory at *Krishi Vigyan Kendra* (KVK) located in the campus of ICAR-Central Institute for Cotton Research (ICAR-CICR), Nagpur in the state of Maharashtra (India). Similarly, the data for Faridkot (Punjab state) were obtained from Central Agricultural Research Station, Faridkot (Punjab Agricultural University, Ludhiana, Punjab). All these stations used automatic weather stations to record daily minimum and maximum temperatures. For remaining four locations viz., Sriganganagar, Rahuri, Lam and Nandyal, the daily data were not available so we used monthly values of minimum and maximum temperatures from Annual reports of ICAR-AICRP on cotton. The time periods of the weather data varied for different locations subjected to availability of weather data itself and also the corresponding data on pink bollworm pheromone trap catches to be used for DD analyses (Table 7).

### Development of degree-day-based phenology model

#### Field calibration and validation of developmental thresholds

Lower and upper developmental temperature thresholds of 13.4 °C and 35.5 °C estimated for pink bollworm in our previous temperature dependent laboratory study (Supplementary information: Annexure I)^16^ were field corrected using a coefficient of variation (CV) technique of degree-day (DD) accumulations^10^. Eight years field data (2009, 2012–2018) on pheromone trap catches of male moths and daily data on minimum and maximum temperatures recorded at Nagpur (Maharashtra) were used for DD accumulation for two events separately: i. DD accumulations started from January 01 of every year till the beginning of moth emergence in that year, and ii. DD accumulations between the consecutive moth peaks starting from beginning of the emergence. A sine wave method with horizontal upper cut-off was used for calculating the DDs between the events as it has been reported to provide DD accumulations with least error across the years or locations^27,28^. Five different combinations of lower and upper thresholds selected for determining field estimates of thresholds were: LTTs- 12.5, 13.0, 13.4, 13.9, 15.5 and HTTs- 32.5, 32.8, 34.0, 35.5, 37.5. The combination of lower and upper developmental thresholds with the lowest CV of DD between events was accepted as the best combination of developmental thresholds to describe the pink bollworm development under field condition (Supplementary information: Annexure II)^10,17,24^. All the calculations of degree days were performed using ‘**DegDay**’ programme, Version 1.01 written in MS-Excel^49^.

The field corrected thresholds were validated as the most appropriate one in terms of providing relatively constant and reliable estimates of DD accumulations between the consecutive moth catch peaks of pink bollworm at three different locations viz., Faridkot, Junagadh, and Dharwad representing north, central and south cotton growing zones of India, respectively. The DD accumulated between the two consecutive moth peaks were adjudged based on the laboratory estimate of 503.62 DD as optimum requirement for completion of one generation of pink bollworm^16^. Accordingly, a value in a range of 500.00 ± 10.00 DD accumulated between any two successive moth peaks was accepted as adequate for pink bollworm development in field condition.

#### Estimation and validation of in-field generation events

For estimating the number of generations, the pink bollworm can complete in a cotton season, a special experiment was planned in the field during two crop seasons of 2018–19 and 2019–20 in the experimental farm of ICAR-Central Institute for Cotton Research (ICAR-CICR), Nagpur. The cotton crop (cultivar: Suraj, non-Bt) was sown on 23 June and 01 July during seasons of 2018–19 and 2019–20, respectively and was maintained by following all the recommended agronomic practices^50^. The foliar spraying of neem oil (Azadirachtin 1500 ppm) @ 3.0 ml per litre of water and flonicamid 50 WG @ 0.4 g per litre of water were given at 45 and 60 days after sowing (DAS) respectively, aiming at controlling the major sucking pest complex of the cotton crop. No any sprays were taken against pink bollworm so as to record its potential infestation during the season.

**Figure 9:**
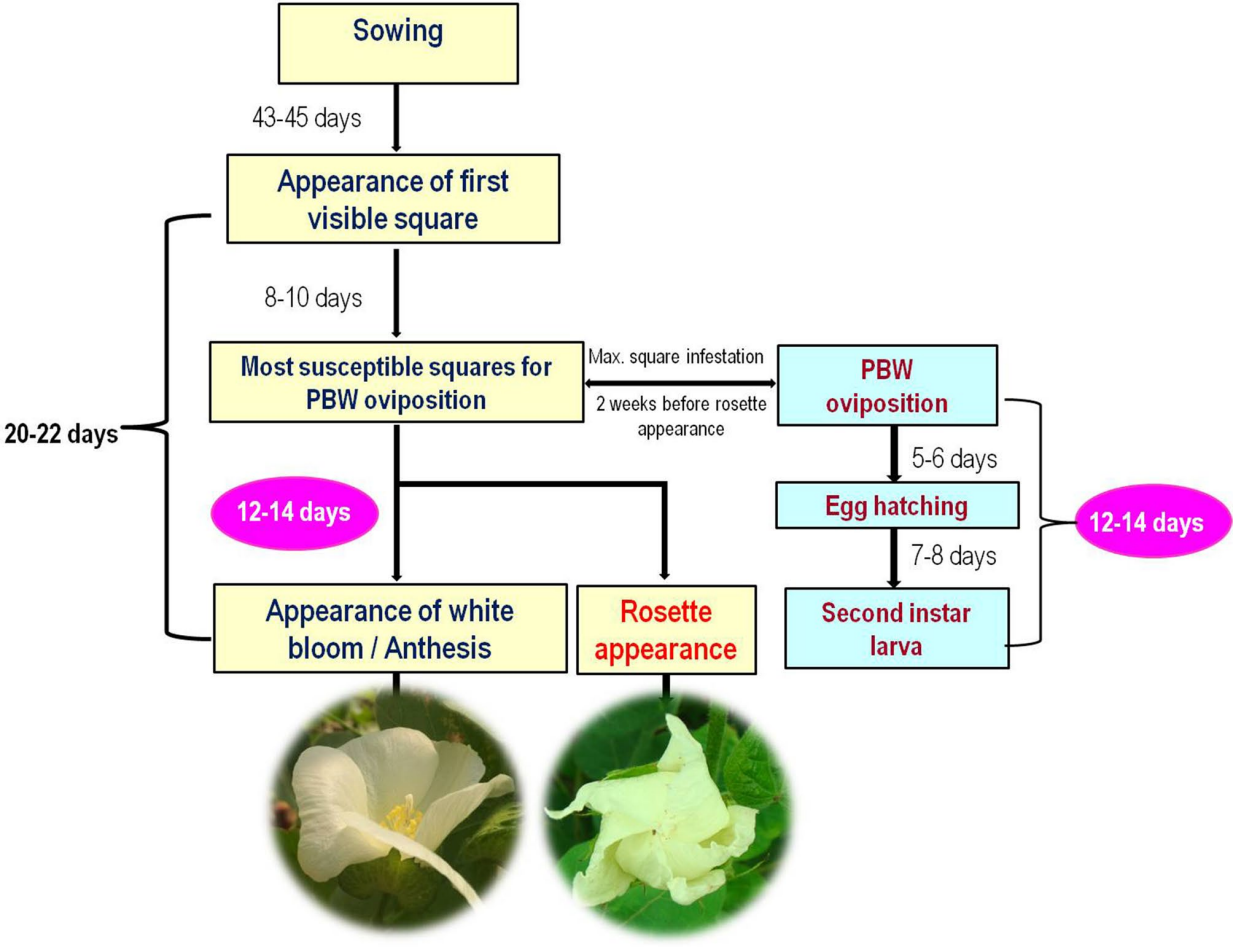
Schematic presentation of a relationship between ‘cotton crop—pink bollworm phenology’ used as a criterion for estimating the generation numbers of pink bollworm based on degree-day accumulations between the successive moth capture peaks obtained in pheromone traps.

Phenological relationship between cotton crop and pink bollworm was taken as criteria for selecting the biofix date for starting of DD accumulation for first in-field generation (F1) (A scheme adopted from Beasley and Adams)^17^. The key basis of host-insect relationship considered here was the link between the number of days from appearance of first square to appearance of first opened flower (anthesis) and the number of days lapsed between oviposition to completion of mid-stage larval development (may be second instar or early third instar) of pink bollworm (Fig. 9). These critical timings of cotton phenology were determined by fixed sampling of 40 cotton plants initially selected randomly, labelled and observed regularly at weekly intervals during both the crop seasons starting from 35 DAS. Each sampled plant was observed for the onset and the number of squares and subsequently for the flowers and the green bolls. Based on the two years numerous observations, the time required for appearance of first just visible square varied from 43–45 DAS. The squares of 8–10 days old are considered the most susceptible for pink bollworm oviposition^17,51^. Our observations also suggested that it usually took 12–14 days from most susceptible square stage to appearance of first open white flower and this time was correlated with the time elapsed between oviposition to mid larval development of pink bollworm^16,52^. This alternately means that if the squares are infested at their most susceptible stage, it will lead to formation of rosette flower 2 weeks later, instead of normal white flower as observed in case of no infestation. Thus, we selected a date 2 weeks prior to rosette formation as a biofix date for initialising degree-day accumulations for the first in-field generation of pink bollworm.

The DD were accumulated between successive moth peaks obtained in data recorded in 30 sex pheromone traps installed along the periphery of cotton fields of ICAR-CICR, Nagpur during 2018–19 and 2019–20. The data of 2018–19 was used in estimation of generation numbers whereas data of 2019–20 was used for its validation. The starting date for DD accumulations for succeeding generation was taken as the peak date of previous generation. If the degree days accumulated between the two consecutive moth peaks using sine wave method with horizontal cut off^27,28^ were in a range of 500.00 ± 10.00 DD, accepted as adequate for completion of one in-field generation of pink bollworm^16^. We also collected a data on rosette flowers and green boll infestation at weekly intervals from 30 m row in each quadrant of the field and were correlated with the male moth captures data in pheromone traps.

### Predictive ability of DD vs ordinal date for predicting pink bollworm phenology

The data set on pink bollworm moth trap catches used in present analysis included a total of 51 surveys conducted over a period of 14 years between 2006 and 2019 at 10 selected sites across three cotton growing zones of India (Table 7). From this data set, we used 51 dates each for beginning of moth emergence and peak moth catches for accumulating DD between these dates for each year at each site. We selected this data based on the following criteria, and for which we had sufficient repeated observations to estimate peak abundance of moth catches. For each combination of site and year, the moth catch data were used in the analysis if, (1) at least two moths per pheromone trap were seen at the site for every observation recorded; (2) there was at least one survey record of pest absence before the emergence date; (3) the insect was seen on more than one survey dates; and (4) there was at least one survey date within two weeks prior to the emergence date^9,17^. Date of first observation of moth catches with ≥ 2 months per trap per week was used as a proxy for emergence date and employed them as biofix dates for starting time series analyses of DD accumulations for all the years at each location. As there were very few occurrences prior to first week of July, we did not use emergence dates that occurred before then.

For each site-year combination, the dates of peak moth catches were determined from the plots of moth trap catches over time. Field calibrated values of 13 °C and 34 °C from present study were used as lower and upper developmental threshold temperatures, respectively. The DD were accumulated between the emergence date and peak dates for each site-year combination using sin wave method with horizontal upper cut off^27,28^. All the 10 selected sites had a minimum of three years of data on moth catches (Table 7), and thus we had at least three years of observed values of peak date and peak DD for each site. At each site, following the alternate exclusion of each year in turn, we calculated the values of mean date and mean DD of peak moth catches from the remaining values. This has provided a unique mean value associated with each observed value. The absolute difference between observed and mean values was taken as an error associated with that variable. To calculate the difference between mean value and observed value of DD on the uniform scale of ordinal date, the mean values for DD were converted back to a date specific to each site and year. A chi-squared test (df = 1) was performed to determine significant difference between DD and date having number of site-year combinations with less error (Supplementary information: Annexure III-A)^9^.

The predictive ability of DD or ordinal dates in forecasting the dates of peak moth emergence was determined by conducting time series analysis using autoregressive integrated moving average (ARIMA) model^53^. From each site, final year of observation was excluded and ARIMA model was applied to the data of remaining years to predict the DD or date of peak moth catches for the last year. This predicted value of peak moth catches was compared with the observed value of that year and difference was worked out as an error associated with it^9^. The ARIMA analysis was performed using XLSTAT-2020 data analysis tool pack in MS-Excel-2010 (Available online at https://www.xlstat.com/en/download, Accessed on 02/04/2020) (Supplementary information: Annexure III-B).

Interannual and latitudinal variations in DD accumulations across different geographical locations were also determined by averaging total accumulated annual GDD among all years at each site. This analysis was performed for only five out of 10 selected locations, due to non-availability of daily temperature data for remaining locations.

## Supplementary Information

Supplementary Information.
