# Author Correction: Degree day-based model predicts pink bollworm phenology across geographical locations of subtropics and semi-arid tropics of India

**DOI:** 10.1038/s41598-021-86714-0

**Published:** 2021-03-29

**Authors:** Babasaheb B. Fand, V. S. Nagrare, S. K. Bal, V. Chinna Babu Naik, B. V. Naikwadi, D. J. Mahule, Nandini Gokte-Narkhedkar, V. N. Waghmare

**Affiliations:** 1 ICAR-Central Institute for Cotton Research, Nagpur, Maharashtra 440 010 India; 2 ICAR-Central Research Institute for Dryland Agriculture, Hyderabad, Telangana 500 059 India

Correction to: *Scientific Reports* https://doi.org/10.1038/s41598-020-80184-6, published online 11 January 2021

This Article contains an error where the state for the location Faridkot is mistakenly given as “Haryana”.

As a result, in the Results section, under the subheading ‘Field calibration and validation of developmental thresholds’,

“The trap catch data used for DD estimation were collected between 2007–16 at Faridkot (Haryana), Junagadh (Gujarat) and Dharwad (Karnataka) locations representing wide variability in diurnal and interannual temperatures from north, central and south cotton growing zones of India.”

should read:

“The trap catch data used for DD estimation were collected between 2007–16 at Faridkot (Punjab), Junagadh (Gujarat) and Dharwad (Karnataka) locations representing wide variability in diurnal and interannual temperatures from north, central and south cotton growing zones of India.”

In Table 2, the cotton growing zone in the header row,

“Faridkot, Haryana”

should read:

“Faridkot, Punjab”

Finally, in Table 6, the state for Faridkot in the North Indian zone,

“Haryana”

should read:

“Punjab”.

