# Supplementary material for: Degree day-based model predicts pink bollworm phenology across geographical locations of subtropics and semi-arid tropics of India
Source: Sci Rep. 2021 Jan 11;11:436. doi: 10.1038/s41598-020-80184-6 (PMC7801711; doi:10.1038/s41598-020-80184-6)
Supplement: Supplementary file 1 — Supplementary Information. [file 41598_2020_80184_MOESM1_ESM.pdf]

Degree day-based model predicts pink bollworm phenology across geographical locations of subtropics and semi-arid tropics of India

Babasaheb B. Fand[1], V.S. Nagrare[1], S.K. Bal[2], V. Chinna Babu Naik[1], B.V. Naikwadi, D. Mahule, Nandini Gokte-Narkhedkar[1], V.N. Waghmare[1]

[1]*ICAR-Central Institute for Cotton Research, Nagpur – 440 010, Maharashtra, India*
[2]*ICAR-Central Research Institute for Dryland Agriculture, Hyderabad – Telangana, India*

Supplementary information

Details of methods and mathematical equations used for performing calculations of degree days, coefficient of variation, Chi2 test, etc.

Annexure I.

Temperature-based phenology model and laboratory based developmental temperature thresholds for pink bollworm

In our previous study (Peddu et al., 2020), we estimated developmental threshold temperatures of 13.4℃/ 35.5℃ (286.45°K/ 308.47°K) and thermal requirements of 503.62 DD for pink bollworm through temperature-dependent phenology model fitted to the laboratory data on its development at different constant temperatures between 15 - 38°C. These laboratory-based thresholds were field-corrected using a coefficient of variation (CV) technique of degree-day (DD) accumulations (Arnold et al., 1959). The model consists of a set of functions that describe the temperature-dependent development in the immature life stages and the senescence in the adult stages. The reproduction was modelled using temperature-dependent oviposition and age-specific fecundity rate. The functions and the parameters used to describe the development, mortality and reproduction are detailed in Table 1. (Peddu et al., 2020).

Table 1. Thermal thresholds and degree day requirements of different developmental stages of pink bollworm based on laboratory study at constant temperatures

Life stage	Linear model*				Sharpe and DeMichele Model							
	Intercept (a)	Slope (b)	Thermal constant (DD)[@]	R2	P	T_0^*	H_a	H_h	$T_h^{\#!}$	$T_I^{\#\%}$	AIC	R2
Egg	-0.1539 (0.0370)[&]	0.0137 (0.0010)	72.99	0.97	0.17 (0.004)*	297.84 (0.00)	16621.42 (0.00)	595843.10 (0.00)	308.43 (0.04)	287.17 (0.90)	- 10.46	0.99
Larvae	-0.0398 (0.0046)	0.0035 (0.0002)	285.71	0.99	0.05 (0.01)	297.74 (0.00)	12032.10 (0.001)	393406.10 (0.00)	308.49 (0.92)	288.181 (0.00)	- 28.70	0.99
Pupa	-0.0759	0.0069	144.92	0.97	0.13 (0.00)	300.28 (1.27)	14309.74 (0.00)	601602.57 (0.00)	308.48 (0.07)	284.00 (0.00)	- 14.61	0.95
**Egg – Adult**	**–**	**–**	**503.62**	**–**	**–**	**–**	**–**	**–**	**308.47 (0.02)**	**286.45 (1.26)**	**–**	**–**

*Test temperature range used for fitting linear relationship: Egg stage (15-35°C); larval stage (15-30 °C) and pupal stage (15 -38 °C)

[&]Numbers in parenthesis are standard errors

[#]Values in °K, to be converted into °C by an equation °C = °K-273

$Theoretical lower development threshold (Tmin), calculated by intercept/slope, ignoring minus sign.

@ Thermal constant calculated by taking inverse of slope (b) *i.e.* 1/b

!Th= Upper temperature threshold (°K)

!Tl= Lower temperature threshold (°K)

Annexure II.

Field calibration and validation of developmental thresholds

Lower and upper developmental temperature thresholds of 13.4°C and 35.5°C estimated for pink bollworm in our previous temperature dependent laboratory study (Peddu et al., 2020) were field corrected using a coefficient of variation (CV) technique of degree-day (DD) accumulations. Eight years field data (2009, 2012-2018) on pheromone trap catches of male moths and daily data on minimum and maximum temperatures recorded at Nagpur (Maharashtra) were used for DD accumulation for two events separately: i. DD accumulations started from January 01 of every year till the beginning of moth emergence in that year, and ii. DD accumulations between the consecutive moth peaks starting from beginning of the emergence. A sine wave method with horizontal upper cut-off was used for calculating the DDs between the events as it has been reported to provide DD accumulations with least error across the years or locations (Allen, 1976; Fry, 1983). Five different combinations of lower and upper thresholds selected for determining field estimates of thresholds were: LTTs- 12.5, 13.0, 13.4, 13.9, 15.5 and HTTs- 32.5, 32.8, 34.0, 35.5, 37.5. The combination of lower and upper developmental thresholds with the lowest CV of DD between events was accepted as the best combination of developmental thresholds to describe the pink bollworm development under field condition (Arnold, 1959; Henneberry and Hutchinson, 1989; Beasley and Adams, 1996).

All the calculations of degree days were performed using '**DegDay.xls**' programme, Version 1.01 written in MS-Excel (Snyder, 2002). Available online at http://biomet.ucdavis.edu/DegreeDays/DegDay.htm. Accessed on 10/01/2018.

The following equations of single sine wave method of degree day estimation were used considering the six possible relationships that exist between the daily temperature cycle and developmental thresholds (Allen, 1976; Zalom et al., 1989).

1. **Daily min and max temperatures are completely above both the thresholds**

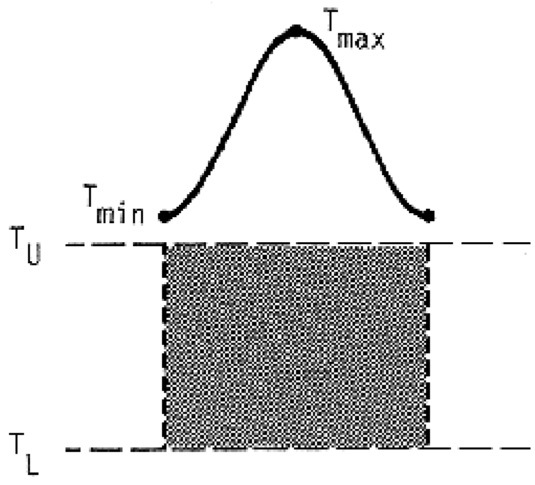

$$°D = T_U - T_L \qquad (1)$$

2. **Daily min and max temperatures are completely below both the thresholds**

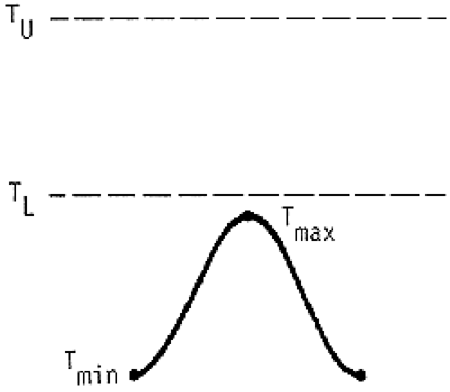

$$°D = 0 \qquad (2)$$

3. **Daily min and max temperatures are entirely between both the thresholds**

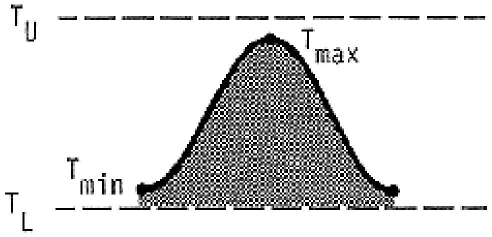

$$°D = \frac{T_{max} + T_{min}}{2} - T_L \qquad (3)$$

4. Min temperature intercepts with lower threshold

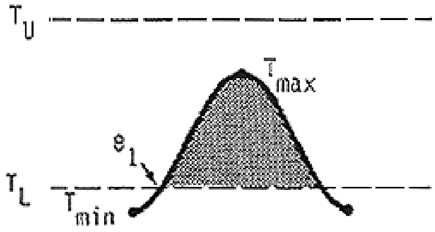

$$°D = \frac{1}{\pi}\left[\left(\frac{T_{max} + T_{min}}{2} - T_L\right)\left(\frac{\pi}{2} - \theta_1\right) + \alpha\cos(\theta_1)\right] \tag{4}$$

$$\theta_1 = \sin^{-1}\left[\left(T_L - \frac{T_{max} + T_{min}}{2}\right) \div \alpha\right]$$

5. Max temperature intercepts with upper threshold

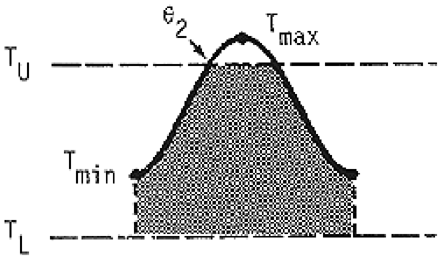

$$°D = \frac{1}{\pi}\left\{\left(\frac{T_{max} + T_{min}}{2} - T_L\right)\left(\theta_2 + \frac{\pi}{2}\right) + \left(T_U - T_L\right)\left(\frac{\pi}{2} - \theta_2\right) - \left[\alpha\cos(\theta_2)\right]\right\} \tag{5}$$

$$\theta_2 = \sin^{-1}\left[\left(T_U - \frac{T_{max} + T_{min}}{2}\right) \div \alpha\right]$$

6. Both min and max temperatures intercepted by both lower and upper thresholds

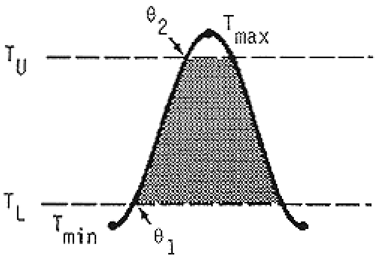

$$°D = \frac{1}{\pi}\left\{\left(\frac{T_{max} + T_{min}}{2} - T_L\right)(\theta_2 - \theta_1) + \alpha[\cos(\theta_1) - \cos(\theta_2)] + (T_U - T_L)\left(\frac{\pi}{2} - \theta_2\right)\right\} \qquad (6)$$

$$\theta_1 = \sin^{-1}\left[\left(T_L - \frac{T_{max} + T_{min}}{2}\right) \div \alpha\right]$$

$$\theta_2 = \sin^{-1}\left[\left(T_U - \frac{T_{max} + T_{min}}{2}\right) \div \alpha\right]$$

Where,

T_U = Upper threshold

T_L = Lower threshold

T_{max} = Maximum temperature

T_{min} = Minimum temperature

$$\alpha = \frac{Tmax - Tmin}{2}$$

Table 1. Degree days estimated from January 01 to beginning of moth emergence using different combinations of lower and upper developmental thresholds for pink bollworm at Nagpur location of Maharashtra state

**Year**	**12.5/ 32.5**	**12.5/ 32.8**	**12.5/ 34**	**12.5/ 35.5**	**12.5/ 37.5**	**13/ 32.5**	**13/ 32.8**	**13/ 34**	**13/ 35.5**	**13/ 37.5**	**13.4/ 32.5**	**13.4/ 32.8**	**13.4/ 34**	**13.4/ 35.5**	**13.4/ 37.5**	**13.9 /32.5**	**13.9/ 32.8**	**13.9/ 34**	**13.9/ 35.5**	**13.9/ 37.5**	**15.5/ 32.5**	**15.5/ 32.8**	**15.5/ 34**	**15.5/ 35.5**	**15.5/ 37.5**
2009	3146	3162.5	3242.6	3325.9	3413.9	3039.4	3061.3	**3141.5**	3224.7	3312.7	2958.6	2980.6	3060.7	3144	3232	2857.9	2879.8	2960	3043.2	3131.2	2537.7	2559.7	2639.8	2723.1	2811.1
2012	3131.1	3148.5	3210.8	3274.5	3338.3	3026.8	3044.1	**3106.4**	3170.1	3233.9	2943.6	2960.9	3023.2	3086.9	3150.7	2840	2857.4	2919.7	2983.4	3047.2	2513	2530.4	2592.7	2656.4	2720.2
2013	3286.7	3303.2	3362.5	3423.8	3487.3	3174.1	3190.6	**3249.9**	3311.1	3374.7	3084.5	3101	3160.3	3221.5	3285.1	2973	2989.5	3048.8	3110.8	3173.6	2620.7	2637.2	2696.5	2757.7	2821.3
2014	3288.3	3306.9	3373.7	3442.6	3511	3177.8	3196.3	**3263.1**	3332.1	3400.4	3089.6	3108.2	3175	3244	3312.3	2979.9	2998.5	3065.3	3134.2	3202.6	2632.9	2651.5	2718.3	2815.3	2855.6
2015	2854	2869.7	2925.1	2977.7	3027.4	2755.5	2771.3	**2826.6**	2879.3	2928.9	2677	2692.8	2748.1	2800.8	2850.4	2579.3	2595	2650.3	2703	2752.7	2270.3	2286	2341.3	2394	2443.6
2016	3077.8	3097.6	3168.8	3241.7	3315.6	2978.5	2998.3	**3069.5**	3142.4	3216.3	2899.5	2919.4	2990.6	3063.5	3137.4	2801.3	2821.2	2892.4	2965.3	3039.2	2490.5	2510.3	2581.5	2654.4	2778.3
2017	3137.4	3155.4	3221.3	3287.4	3355.2	3032.5	3050.2	**3116.1**	3182.1	3250	2948.5	2966.6	3032.5	3098.5	3166.3	2844.7	2862.7	2928.6	2994.7	3062.5	2518.6	2536.6	2602.5	2668.6	2736.4
2018	2816.5	2833.6	2895.3	2958.6	3022.2	2722.6	2739.7	**2815.3**	2864.7	2928.3	2647.8	2664.9	2726.6	2789.5	2853.5	2554.8	2571.9	2633.5	2696.9	2760.5	2261.4	2278.5	2340.2	2403.5	2437.1
SUM	24737.8	24877.4	25400.1	25932.2	26470.9	23907.2	24051.8	**24588.4**	25106.5	25645.2	23249.1	23394.4	23917	24448.7	24987.7	22430.9	22576	23098.6	23631.5	24169.5	19845.1	19990.2	20512.8	21073	21603.6
**Mean**	**3092.22**	**3109.67**	**3175.01**	**3241.52**	**3308.86**	**2988.40**	**3006.47**	**3073.55**	**3138.31**	**3205.65**	**2906.14**	**2924.30**	**2989.62**	**3056.09**	**3123.46**	**2803.86**	**2822.00**	**2887.32**	**2953.94**	**3021.19**	**2480.64**	**2498.77**	**2564.10**	**2634.12**	**2700.45**
**SD**	**175.54**	**175.94**	**178.63**	**182.68**	**188.30**	**169.61**	**170.21**	**169.97**	**177.19**	**182.97**	**165.06**	**165.72**	**168.62**	**172.98**	**178.80**	**159.44**	**160.15**	**163.19**	**167.66**	**173.61**	**141.90**	**142.71**	**146.09**	**155.36**	**166.47**
**CV**	**5.677**	**5.658**	**5.626**	**5.636**	**5.691**	**5.676**	**5.662**	**5.530**	**5.646**	**5.708**	**5.680**	**5.667**	**5.640**	**5.660**	**5.724**	**5.687**	**5.675**	**5.652**	**5.676**	**5.747**	**5.720**	**5.711**	**5.698**	**5.898**	**6.165**

Table 2. Field calibration of optimum developmental threshold temperatures for cotton pink bollworm using coefficients of variation (CV) technique of degree-day accumulations calculated for male moth catches in sex pheromone traps observed for a period of 8 years using different combinations of lower and upper threshold temperatures. A combination with lowest CV is accepted as the best lower and upper threshold temperatures.

January 01 to beginning of moth emergence					
**UTTs →** / **LTTs ↓**	**32.5**	**32.8**	**34.0**	**35.5**	**37.5**
**12.5**	5.68	5.66	5.63	5.64	5.69
**13.0**	5.68	5.66	**5.53**	5.65	5.71
**13.4**	5.68	5.67	5.64	5.66	5.72
**13.9**	5.69	5.68	5.65	5.68	5.75
**15.5**	5.72	5.71	5.70	5.90	6.16

$$\text{Coefficient of variation} = \frac{\text{Standar deviation}}{\text{Mean}} \text{ x } 100 \qquad (7)$$

Annexure III

Predictive ability of DD vs ordinal date for predicting pink bollworm phenology

A. *Determination of Degree days and dates to peak moth emergence:*

For each site-year combination, the dates of peak moth catches were determined from the plots of moth trap catches over time. Field calibrated values of 13℃ and 34℃ from present study were used as lower and upper developmental threshold temperatures, respectively. The DD were accumulated between the emergence date and peak dates for each site-year combination using sin wave method with horizontal upper cut off (Allen, 1976; Fry, 1983). All the 10 selected sites had a minimum of three years of data on moth catches (Table 2), and thus we had at least three years of observed values of peak date and peak DD for each site. At each site, following the alternate exclusion of each year in turn, we calculated the values of mean date and mean DD of peak moth catches from the remaining values. This has provided a unique mean value associated with each observed value. The absolute difference between observed and mean values was taken as an error associated with that variable. To calculate the difference between mean value and observed value of DD on the uniform scale of ordinal date, the mean values for DD were converted back to a date specific to each site and year. A chi-squared test (df =1) was performed to determine significant difference between DD and date having number of site-year combinations with less error (Cayton et al., 2015).

The detailed calculations for Nagpur (Maharashtra state) locations are provided as an example. Similar procedure was followed for all remaining locations.

i. Degree days to peak emergence

**Year**	**Observed DD**	**Mean DD***	**Difference in DD (Error)**
2009	1519.3	1518.27	1.03
2012	1521	1518.03	2.97
2013	1511	1519.46	-8.46
2014	1517.2	1518.57	-1.37
2015	1521.5	1517.96	3.54
2016	1519.3	1518.27	1.03
2017	1518.4	1518.40	0
2018	1519.5	1518.24	1.26

$$* \boldsymbol{Mean\ DD} = \frac{\sum \boldsymbol{Observed\ DD\ of}(\boldsymbol{n-1})}{(\boldsymbol{n-1})} \quad \textbf{(8)}$$

(n-1) is followed for alternate exclusion of each year from total number of years and calculating the mean value of DD from DD values of remaining years

B. e.g. for year, 2009, the mean DD value was calculated by averaging observed ***values*** of remaining years.

$$\text{Mean DD} = \frac{\sum 1521 + 1511 + 1517.2 + 1521.5 + 1519.3 + 1518.4 + 1519.5}{7}$$

= 1518.27

Same procedure was repeated for all the years.

ii. Dates to peak emergence (ordinal dates observed)

Year	Observed date	Mean date*	Difference in days
2009	02-Dec	06-Dec	-4.86
2012	16-Dec	04-Dec	11.14
2013	09-Dec	05-Dec	3.14
2014	02-Dec	06-Dec	-4.86
2015	25-Nov	07-Dec	-12.86
2016	08-Dec	06-Dec	2.00
2017	02-Dec	06-Dec	-4.86
2018	16-Dec	04-Dec	11.14

*Mean dates were calculated by same way as that of mean DD explained above

iii. Mean dates converted from mean DD

**Year**	Mean DD	Mean Dates converted from mean DD
2009	1518.27	02-Dec
2012	1518.03	16-Dec
2013	1519.46	10-Dec
2014	1518.57	02-Dec
2015	1517.96	25-Nov
2016	1518.27	08-Dec
2017	1518.4	02-Dec
2018	1518.24	16-Dec

iv. Difference of observed and mean dates

Year	Observed date (ordinal)	Mean dates converted from DD	Difference in days
2009	02-Dec	02-Dec	0.00
2012	10-Dec	16-Dec	-6.00
2013	09-Dec	10-Dec	-1.00
2014	02-Dec	02-Dec	0.00
2015	25-Nov	25-Nov	0.00
2016	09-Dec	08-Dec	1.00
2017	03-Dec	02-Dec	1.00
2018	16-Dec	16-Dec	0.00

v. Absolute difference (error) between observed and predicted values from DD converted to dates and ordinal dates

<table>
<tr><th rowspan="2">Year</th><th rowspan="2">Difference in days (converted from DD)</th><th rowspan="2">Difference in days (ordinal dates)</th><th colspan="2">Number of years with less error</th><th>Chi-2 (df = 1)</th><th>P value</th></tr>
<tr><th>DD</th><th>Date</th><td rowspan="9">1.8</td><td rowspan="9">0.179</td></tr>
<tr><td>2009</td><td>0.00</td><td>-4.86*</td><td rowspan="8">08</td><td rowspan="8">05</td></tr>
<tr><td>2012</td><td>-6.00</td><td>11.14</td></tr>
<tr><td>2013</td><td>-1.00</td><td>3.14*</td></tr>
<tr><td>2014</td><td>0.00</td><td>-4.86*</td></tr>
<tr><td>2015</td><td>0.00</td><td>-12.86</td></tr>
<tr><td>2016</td><td>1.00</td><td>2.00*</td></tr>
<tr><td>2017</td><td>1.00</td><td>-4.86*</td></tr>
<tr><td>2018</td><td>0.00</td><td>11.14</td></tr>
</table>

*The years with error value < 5 days were treated as years with less error

B. Time series analysis using ARIMA model

The predictive ability of DD or ordinal dates in forecasting the dates of peak moth emergence was determined by conducting time series analysis using autoregressive integrated moving average (ARIMA) model (Box and Jenkin, 1970). From each site, final year of observation was excluded and ARIMA model was applied to the data of remaining years to predict the DD or date of peak moth catches for the last year. This predicted value of peak moth catches was compared with the observed value of that year and difference was worked out as an error associated with it (Cayton et al., 2015). The ARIMA analysis was performed using XLSTAT-2020 data analysis tool pack in MS-Excel-2010 (Available online at https://www.xlstat.com/en/download, Accessed on 02/04/2020).

Eight years data from Nagpur location was explained here as an example. The similar procedure was used for remaining locations to construct the ARIMA model.

Year	Observed DD to peak emergence
2009	1519.3
2012	1521
2013	1511
2014	1517.2
2015	1521.5
2016	1519.3
2017	1518.4
**2018**	**1519.5***

This year is excluded from analysis and the predictions are made from only seven years' data points. The value predicted for the year 2018 was compared with predicted value for the same year using ARIMA model

The steps of solving ARIMA model using XLSTAT-2020 data analysis tool pack in MS-Excel-2010 is detailed below:

Model parameters: p = 1 / d = 1 / q = 1 / P = 0 / D = 0 / Q = 0 / s = 0

Optimize: Likelihood (Convergence = 0.00001 / Iterations = 500)

Validation: 2

Prediction: 1

Confidence intervals (%): 95

Summary statistics:

Variable	Observations	Obs. with missing data	Obs. without missing data	Minimum	Maximum	Mean	Std. deviation
Yt	7	0	7	1511.00	1521.50	1518.24	3.51

Results of ARIMA modelling of the Yt series:

Goodness of fit statistics:

Observations	4
DF	1
SSE	72.84879
MSE	18.2122
RMSE	4.267575
WN Variance	18.2122
MAPE(Diff)	58.04669
MAPE	0.165495
-2Log(Like.)	24.55222
FPE	30.35366
AIC	30.55222
AICC	
SBC	28.7111
Iterations	14

Model parameters:

Parameter	Value	Hessian standard error	Lower bound (95%)	Upper bound (95%)
Constant	0.066	1.355	-2.590	2.723

Parameter	Value	Hessian standard error	Lower bound (95%)	Upper bound (95%)	Asympt. standard error	Lower bound (95%)	Upper bound (95%)
AR(1)	0.011	0.579	-1.124	1.145	0.500	-0.969	0.991
MA(1)	-1.000	0.740	-2.451	0.451	0.000	-1.000	-1.000

Predictions and residuals:

Observations	Yt	ARIMA(Yt)	Residuals	Standardized residuals	Standard error	Lower bound (95%)	Upper bound (95%)
2009	1519.300	1519.300	0.000	0.000			
2012	1521.000	1519.839	1.161	0.272			
2013	1511.000	1518.573	-7.573	-1.775			
2014	1517.200	1517.159	0.041	0.010			
2015	1521.500	1517.738	3.762	0.881			
2016	1519.300	1518.533	0.767	0.180	4.268	1510.168	1526.897
2017	1518.400	1518.501	-0.101	-0.024	4.268	1510.136	1526.866
**2018**		**1518.501**			4.268	1510.136	1526.865

Descriptive analysis (Yt):

Lag	Autocorrelation	Standard error	Lower bound (95%)	Upper bound (95%)	Partial autocorrelation	Standard error	Lower bound (95%)	Upper bound (95%)
0	1.000	0.000			1.000	0.000		
1	-0.125	0.378	-0.741	0.741	-0.125	0.378	-0.741	0.741
2	-0.469	0.384	-0.752	0.752	-0.493	0.378	-0.741	0.741
3	0.001	0.458	-0.899	0.899	-0.200	0.378	-0.741	0.741
4	0.071	0.458	-0.899	0.899	-0.283	0.378	-0.741	0.741
5	0.021	0.460	-0.902	0.902	-0.185	0.378	-0.741	0.741
6	0.002	0.460	-0.902	0.902	-0.194	0.378	-0.741	0.741
7	0.000	0.460	-0.902	0.902	-0.154	0.378	-0.741	0.741
8	0.000	0.460	-0.902	0.902	-0.147	0.378	-0.741	0.741

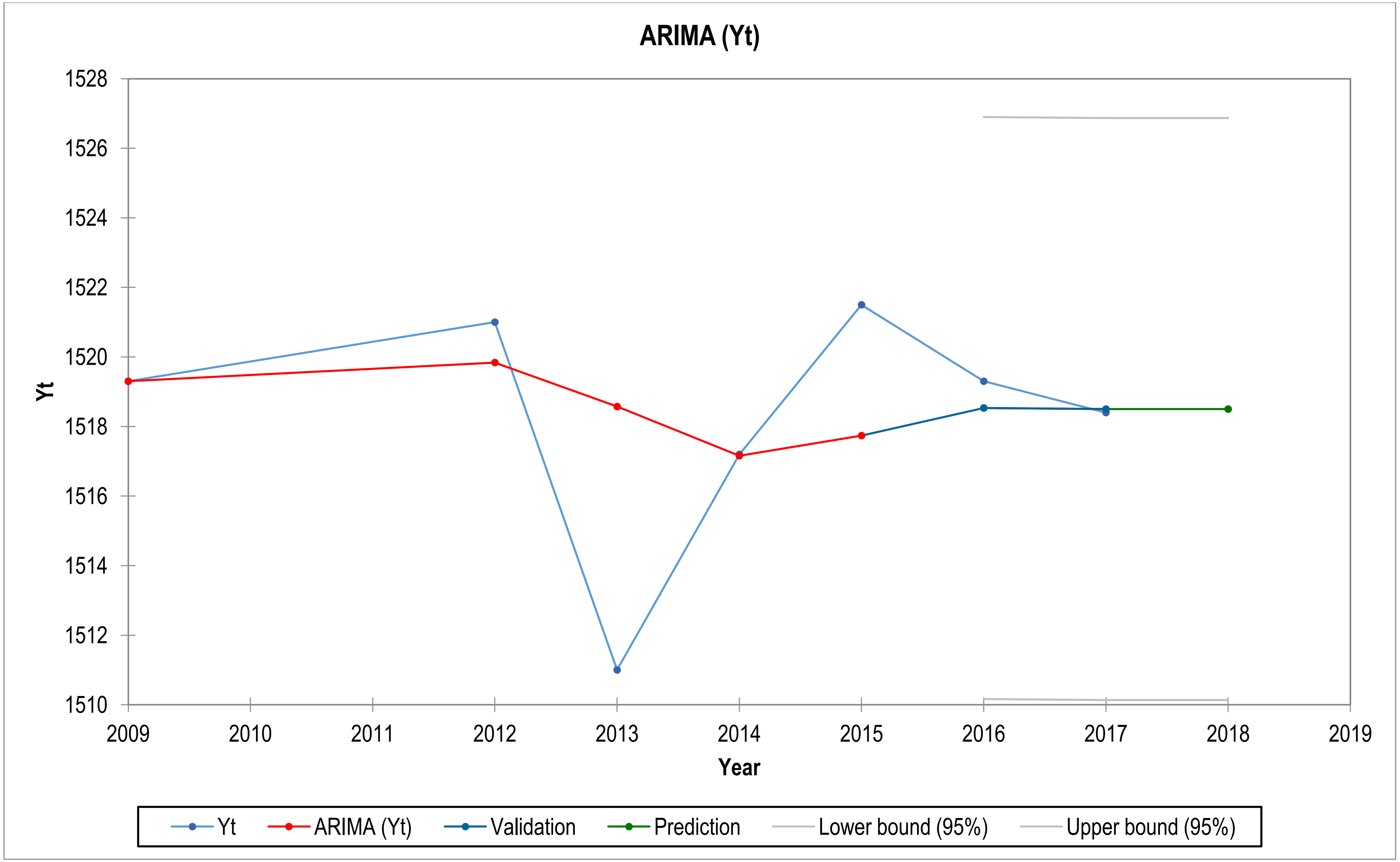

ARIMA (Yt)
1528
1526
1524
1522
1520
1518
1516
1514
1512
1510
Yt
2009
2010
2011
2012
2013
2014
2015
2016
2017
2018
2019
Year
Yt
ARIMA (Yt)
Validation
Prediction
Lower bound (95%)
Upper bound (95%)

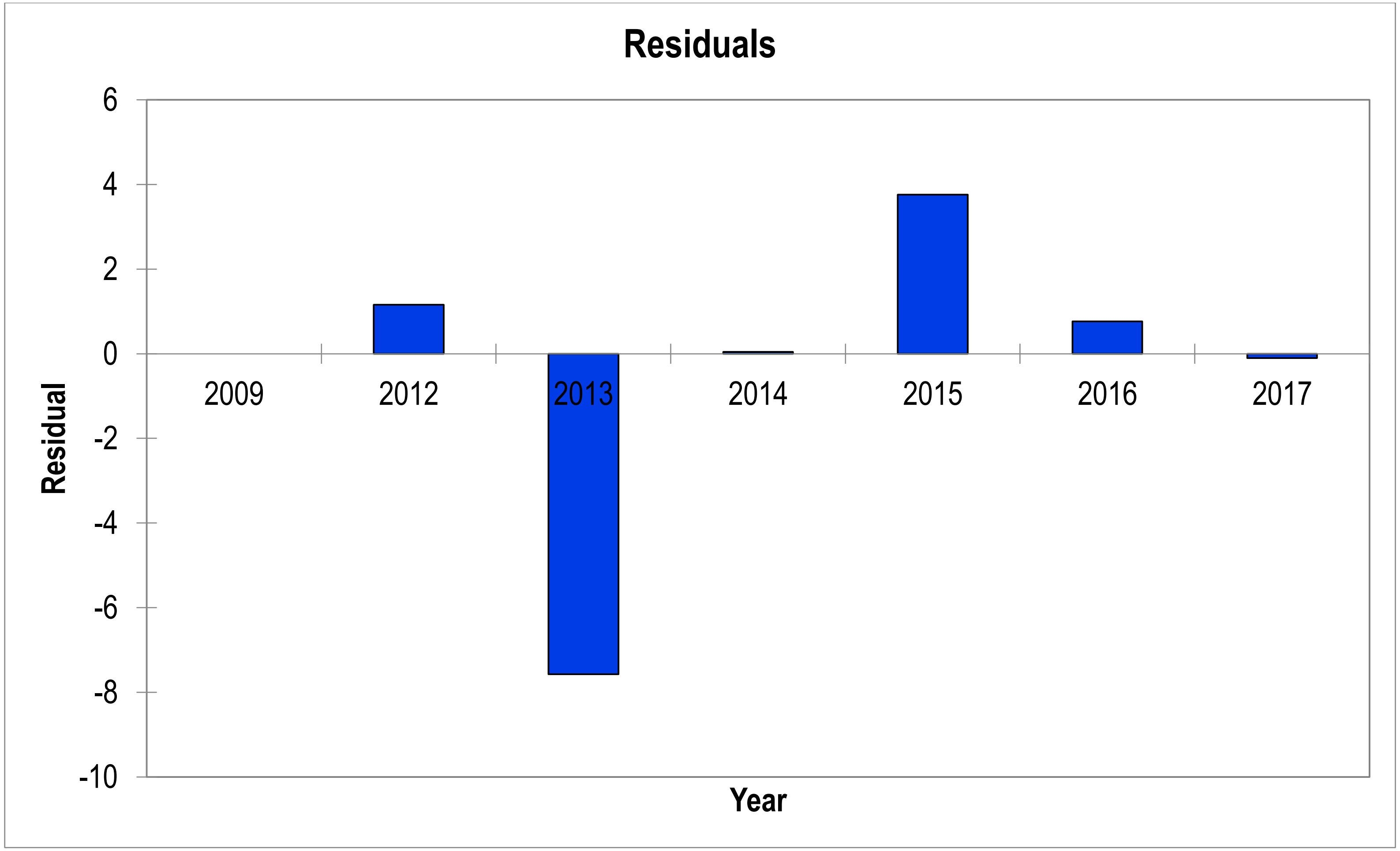
Residuals
Residual
6
4
2
0
-2
-4
-6
-8
-10
2009
2012
2013
2014
2015
2016
2017
Year

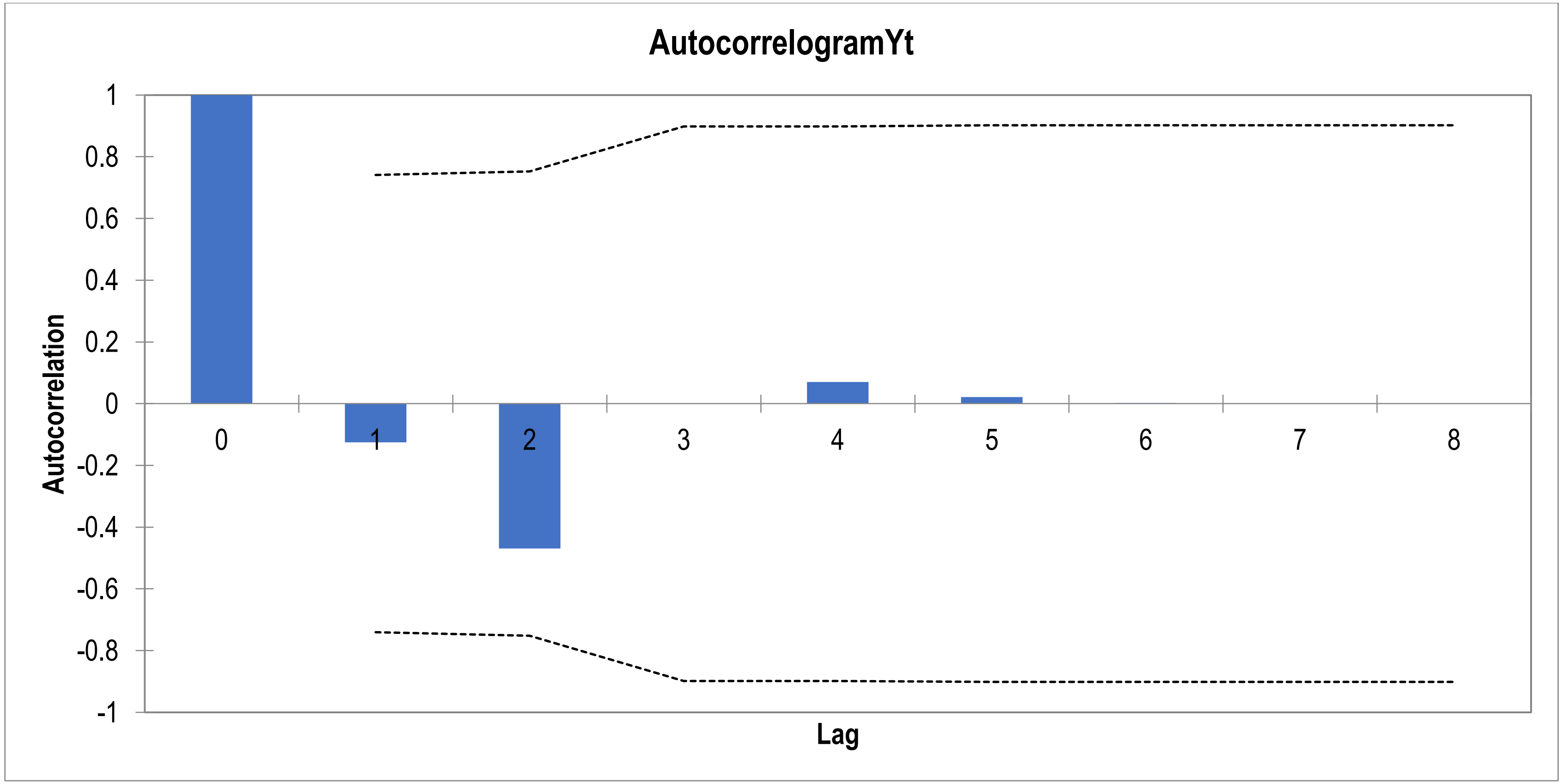

AutocorrelogramYt
Autocorrelation
1
0.8
0.6
0.4
0.2
0
-0.2
-0.4
-0.6
-0.8
-1
0
1
2
3
4
5
6
7
8
Lag

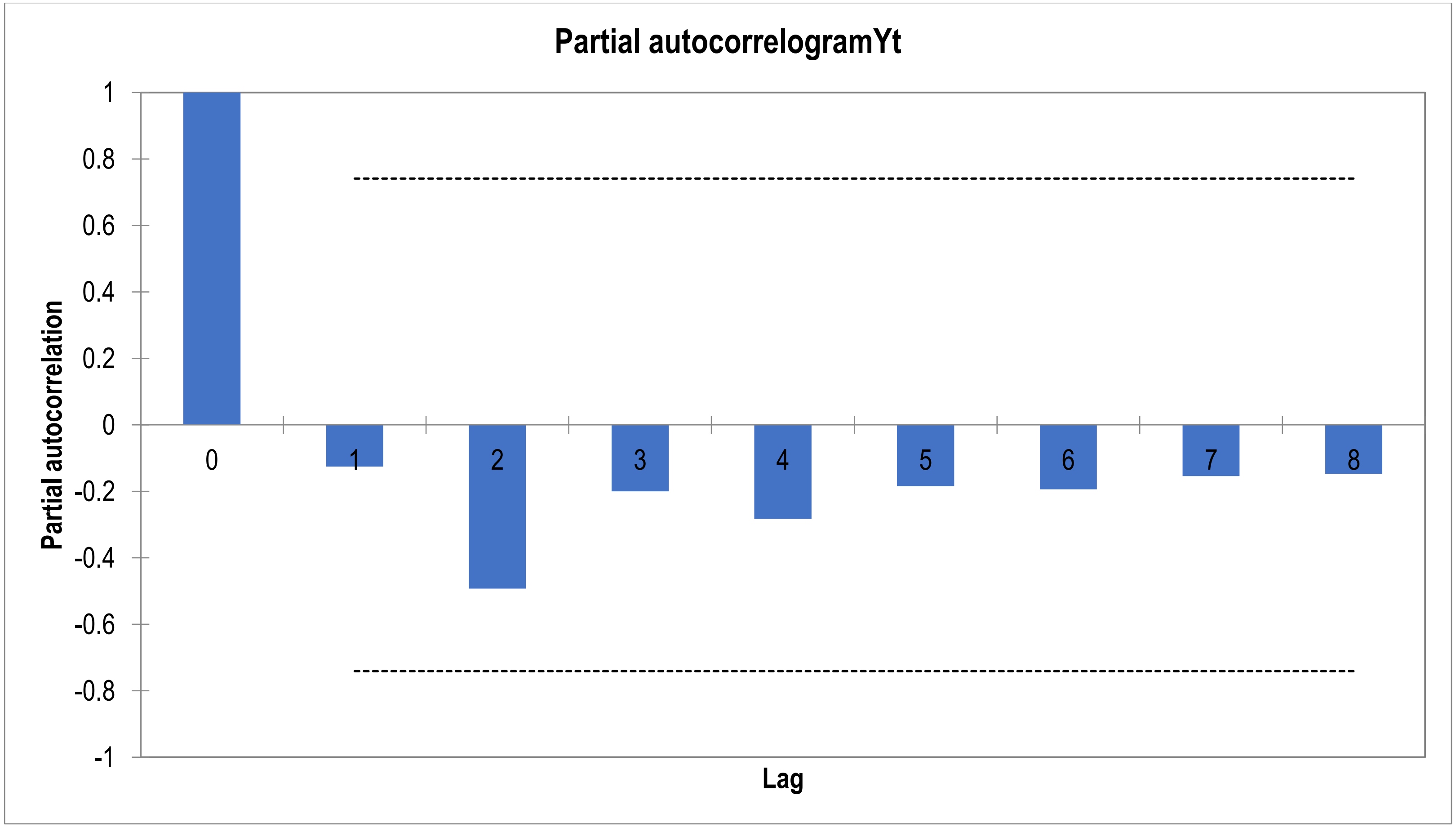
Partial autocorrelogramYt
Partial autocorrelation
1
0.8
0.6
0.4
0.2
0
-0.2
-0.4
-0.6
-0.8
-1
0
1
2
3
4
5
6
7
8
Lag
